# *Platycodon grandiflorum* Triggers Antitumor Immunity by Restricting PD-1 Expression of CD8^+^ T Cells in Local Tumor Microenvironment

**DOI:** 10.3389/fphar.2022.774440

**Published:** 2022-04-14

**Authors:** Ruijie Yang, Tianli Pei, Ruifei Huang, Yue Xiao, Jiangna Yan, Jinglin Zhu, Chunli Zheng, Wei Xiao, Chao Huang

**Affiliations:** ^1^ Xi’an International Medical Center Hospital Affiliated to Northwest University, Xi’an, China; ^2^ Key Laboratory of Resource Biology and Biotechnology in Western China (Northwest University), Ministry of Education, School of Life Sciences, Northwest University, Xi’an, China; ^3^ State Key Laboratory of New-tech for Chinese Medicine Pharmaceutical Process, Jiangsu Kanion Parmaceutical, Co, Ltd., Lianyungang, China; ^4^ Lab of Systems Pharmacology, Center of Bioinformatics, College of Life Science, Northwest A&F University, Yangling, China

**Keywords:** *Platycodon grandiflorum*, systems pharmacology, CD8^+^ T cells, tumor microenvironment, VEGF-A–VEGFR2

## Abstract

In the tumor microenvironment (TME), the activation of programmed death-1 (PD-1)–programmed death ligand-1 (PD-L1) pathway is one of the main signals of immune escape and tumor deterioration. Clinically, the application of monoclonal antibodies slows down the progression of various malignancies and prolongs the survival of patients effectively. However, these treatments result in serious immune-related adverse events (irAEs) owning to systemic immune activation. Therefore, to achieve long-term therapeutic effects and low side effects, it is necessary to find drugs inhibiting the local PD-1/PD-L1 signaling pathway of the TME. Here, we discovered that *Platycodon grandiflorum* (PG), a medicine and food homology herb, reduced the expression of PD-1 on the surface of CD8^+^ T cells to exert antitumor effects in non-small cell lung cancer (NSCLC). Firstly, by combining systems pharmacology strategies and clinical data analysis, we found that PG has the potential to immunomodulate T cells and suppress tumors. Secondly, *in vivo* and *in vitro* experiments have confirmed the antitumor effect of the combination of Platycodin D and Platycodin D3, which is preferred and representative of the compounds. Mechanistically, PG increased the infiltration and killing activity of CD8^+^ T cells, which was related to the decrease of PD-1^+^ CD8^+^ T cells. Furthermore, we confirmed that PG regulated the expression of PD-1 on the surface of CD8^+^ T cells *via* reducing the secretion of VEGF-A regulated by the level of P-STAT3 in tumor cells. Additionally, PG also positively impacted the biological processes downstream of STAT3. Overall, we demonstrated that PG-mediated downregulation of PD-1 on the surface of CD8^+^ T cells represents a promising strategy to locally enhance T-cell responses and improve antitumor immunity.

## Introduction

Programmed death-1 (PD-1) is an inhibitory receptor from the CD28 family and has attracted a substantial amount of attention in the field of cancer research in recent years. It is expressed on various immune cells, namely, peripherally activated T and B lymphocytes, dendritic cells (DCs), monocytes, natural killer (NK), and macrophages (Agata et al., 1996; Yamazaki et al., 2002; Keir et al., 2008). Although there is a relatively wide expression pattern for PD-1, its most important role is likely as a co-inhibitory receptor on T cells (Chikuma, 2016; Wartewig and Ruland, 2019). When binding to its ligand (mainly programmed death ligand-1, PD-L1), PD-1 can activate intracellular signaling pathways and inhibit the activation of T cells, thereby reducing the secretion of cytokines and even depleting T cells, avoiding excessive immune responses and autoimmunity (Sharpe et al., 2007; Francisco et al., 2010; Pedoeem et al., 2014). Nevertheless, PD-L1 also shows an abnormally high expression in tumor cells (Mu et al., 2011; Lin et al., 2015; Qin et al., 2015; Zhou et al., 2017), which is considered the main factor responsible for promoting the ability of tumor immune escape (Jiang et al., 2019). The PD-1/PD-L1 signaling pathway is an important component of tumor immunosuppression, which inhibits the activation of T lymphocytes and enhances the immune tolerance of tumor cells, thereby achieving tumor immune escape. Therefore, targeting the PD-L1/PD-1 pathway is an attractive strategy for cancer treatment (Zou et al., 2016).

Current therapeutic approaches focus on blocking the interaction of this receptor with its ligands to enhance T-cell responses (Ohaegbulam et al., 2015). The drugs targeting PD-1 in patients had led to long-lasting tumor responses and restoration of antitumor immunity, especially monoclonal antibodies, such as nivolumab and pembrolizumab. They have revolutionized the treatment of melanoma and are currently being evaluated as a treatment for a wide range of other cancers, including NSCLC, renal cell carcinoma, ovarian cancer, and Hodgkin lymphoma (Topalian et al., 2012; Westin et al., 2014; Hamanishi et al., 2015; Patnaik et al., 2015). Although the PD-1 monoclonal antibody brings great hope to cancer patients, targeted therapy also has serious irAEs. IrAEs of PD-1 inhibition are most commonly observed in the skin, gastrointestinal tract, liver, and endocrine systems and include pruritus, rash, nausea, diarrhea colitis, pneumonitis, thyroid disorders, and lung toxicities (Boutros et al., 2016; Wang et al., 2019; Ramos-Casals et al., 2020). The reason for these symptoms is because the PD-1 monoclonal antibody changes the entire immune state of the immune system and not just the tumor microenvironment (TME) (Michot et al., 2016; Darvin et al., 2018). Based on these, the development of drugs capable of targeting the TME and having low side effects is becoming a real challenge that is currently addressed (Roma-Rodrigues et al., 2019; Schirrmacher, 2019).

*Platycodon grandiflorum* (PG), a medicine and food homology traditional Chinese medicinal herb, has been widely used as a traditional Asian medicine for the treatment of pulmonary and respiratory disorders (Zhang et al., 2015). In recent years, PG and some of the major components of PG, especially Platycodin D (PD), have been found to have diverse pharmacological activities, namely, anti-inflammatory (Wang et al., 2016; Meng et al., 2017; Ye et al., 2019), anti-obesity, and hyperlipidemia effects (Zhao et al., 2006; Lee et al., 2011), antioxidant and antimicrobial activities (Sheng et al., 2017), multiple protective effects on the liver (Wu et al., 2016; Leng et al., 2018; Choi et al., 2020; Liu et al., 2020), and antitumor effects. Furthermore, some studies have shown that PG and its main components have immunomodulatory effects, such as regulating macrophage activity (Wang et al., 2004), mast cell inflammatory response (Han et al., 2009), and DC maturation (Park et al., 2014). In terms of antitumor, most studies have reported that they exert potent growth inhibition, strong cytotoxicity against various cancer cell lines, and robust antiangiogenic activity on endothelial cells (Kim et al., 2008; Lee et al., 2008; Chun et al., 2013; Chun and Kim, 2013; Luan et al., 2014; Xu et al., 2014; Zhao et al., 2015; Li et al., 2016a; Fu et al., 2017; Zhang et al., 2017). Additionally, they significantly inhibit tumor growth in mice bearing liver cancer cells (Li et al., 2015a; Li et al., 2016b). The molecular mechanisms responsible for the anticancer activity involve the suppression of Akt, PI3K, MAPK, JNK, ROS, NF-KB, and other pathways, promotion of apoptosis and inhibition of cell cycle, and proliferation. Even though research on PG and the major components has become increasingly in-depth, it remains unknown whether PG enables to achieve antitumor effects through immune regulation, as well as the mechanism(s) of how PG promotes antitumor immunity.

Here, we show that PG reduces the expression of PD-1 on the surface of CD8^+^ T cells to exert antitumor effects in NSCLC. Specifically, we first predicted the potential antitumor effects of PG by systems pharmacology and clinical data analysis. Simultaneously, we selected the combination of PD and *PlatycodinD3* (PD3) with the highest content and stronger pharmacokinetic activity in PG through pharmacokinetic prediction, as the effective component of PG for subsequent research. We found that PG has a significant inhibitory effect on a variety of tumor cell lines, and the Lewis lung cancer (LLC) cell line is the most sensitive to its effect. We verified the effect of PG on LLC tumor-bearing mice, and it had indeed markedly slowed down tumor growth and prolonged survival. In addition, based on the systems pharmacology analysis and flow cytometry analysis, we noticed that PG reduces the infiltration of PD-1^+^ CD8^+^ T cells to release antitumor activity. Mechanistically, we determined that the reduction of PD-1 is related to the VEGF-A–VEGFR-2 axis which is indirectly regulated by PG attenuating the secretion of VEGF-A derived from tumor cells, and we further proved that the reduction of VEGF-A is due to the downregulation of the phosphorylation of STAT3. In addition, we also found that PG positively adjusts the biological processes downstream of STAT3: apoptosis, cell cycle, and proliferation. In conclusion, our results indicate the fact that PG-mediated downregulation of PD-1 on the surface of CD8^+^ T cells provides a significant strategy for immunotherapy.

## Results

### Potential Antitumor Effects of *Platycodon grandiflorum*

To reveal the potential antitumor mechanisms of PG at a system level, we firstly collected its chemical components (Supplementary Table S1) by extracting from our database Traditional Chinese Medicine Systems Pharmacology (TCMSP) (Ru et al., 2014) and using manual literature mining. To further obtain potential active compounds from PG, we calculated and screened out 30 potential active compounds (Supplementary Table S2), which were considered “candidate compounds”, from PG *via* the ADME parameters of oral bioavailability (OB), drug-likeness (DL), or half-life period (HL) according to our previous study (Liu et al., 2016; Zheng et al., 2021). We observed that the PD which was reported had the highest content in PG-possessing optimal pharmacokinetic parameters (HL = long). We also tested the content of the three most reported compounds in the extract by HPLC and found that the content of Platycodin D is also the highest (Supplementary Figure S2A). We then predicted the potential targets for PG by utilizing the weighted ensemble similarity (WES) and systematic drug targeting tool (Sys DT) methods which can synthetically obtain the direct and indirect target information for PG. Eventually, we got 175 nonredundant targets for PG (Supplementary Table S3).

**FIGURE 1:**
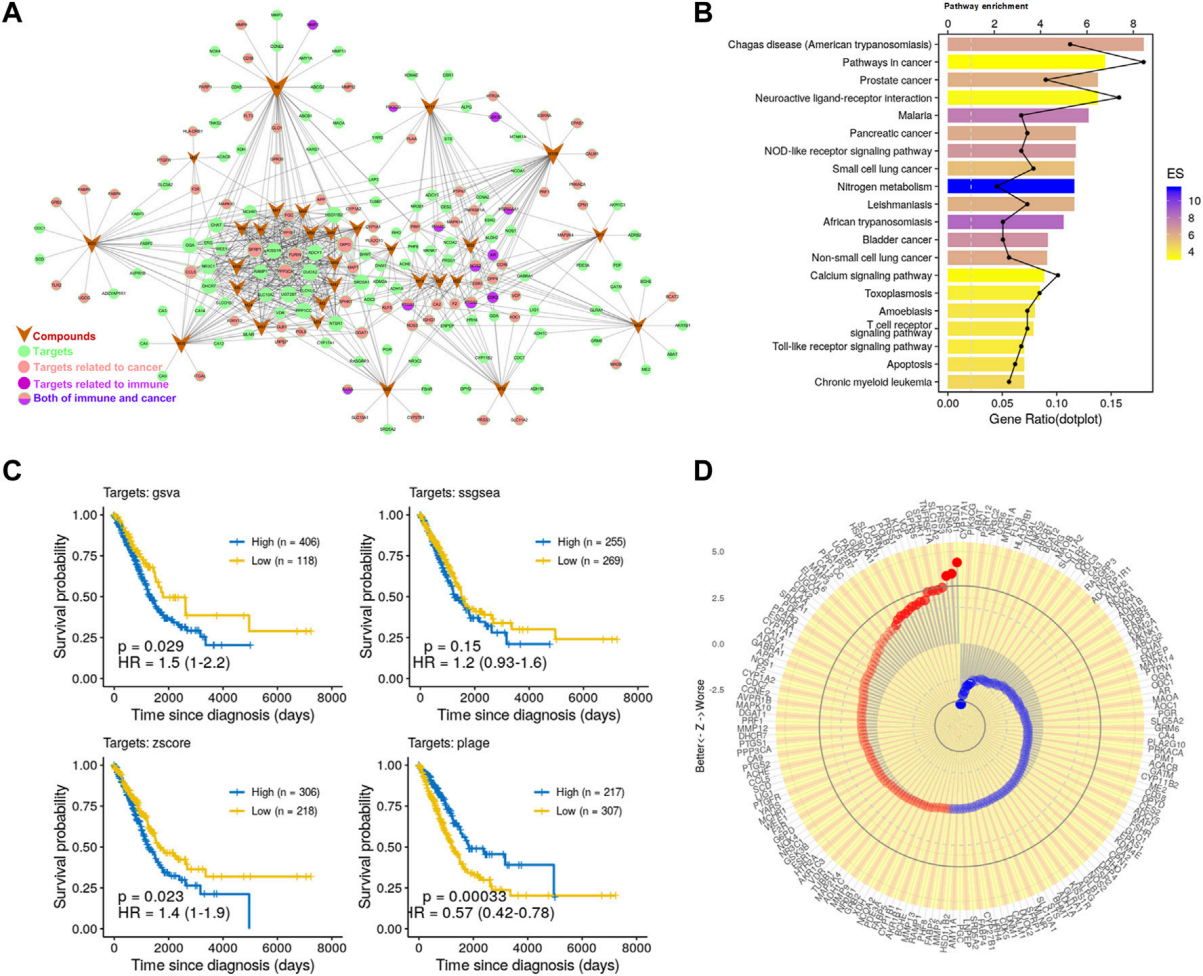
Potential antitumor effects of PG. **(A)** C-T network. The meanings of colors and shapes are shown in the figure. The circle represents the target corresponding to the active compound, V-shape represents active compounds, and edge represents the relationship between the active compound and target. **(B)** The KEGG pathway enrichment of potential targets. **(C)** Kaplan–Meier curves of LUAD visualize the relationship between targets and overall survival of LUAD patients comparing high and low activities. These four Kaplan–Meier curves were evaluated by the four gene set enrichment analysis approaches, respectively. **(D)** Association of the 175 targets of PG with patient survival. Z value was obtained from the Cox regression model.

To visualize the relationship between these targets and compounds, the C-T network graph (Figure 1A) was constructed for the 30 compounds and 175 targets through 648 interactions. The network topology analysis results in the average degree of each compound and each protein are 21.56 and 3.7, respectively. Notably, the degree of PD (M22, degree = 22) and PD3 (M24, degree = 24) is shown as relatively high among the represent active compounds, indicating its potential for medicinal use, and they are the most abundant ingredients (Supplementary Figure S2A). Based on these, we used a combination of PD and PD3 (1.5:1) as a representative drug of PG for cell-level and animal-level verifications. These results indicate the potential drug ability of PG, PD3, and PD. Consistently, PG, PD3, and PD have been reported to have robust pharmacological activities (Zhao et al., 2006; Kim et al., 2008; Lee et al., 2008; Lee et al., 2011; Chun et al., 2013; Chun and Kim, 2013; Luan et al., 2014; Xu et al., 2014; Zhao et al., 2015; Li et al., 2016a; Li et al., 2016b; Wang et al., 2016; Wu et al., 2016; Fu et al., 2017; Meng et al., 2017; Sheng et al., 2017; Zhang et al., 2017; Leng et al., 2018; Ye et al., 2019; Choi et al., 2020; Liu et al., 2020).

Next, in order to detect whether these targets are associated with cancer, we further enriched these pathways (Figure 1B). We found that the significantly enriched pathways mainly involve cancer and immunity. Such as the “Pathways in cancer”, “Prostate cancer”, “Non-small cell lung cancer,” and other cancer-related pathways and the “NOD-like receptor signaling pathway”, “T cell receptor signaling pathway”, “Toll-like receptor signaling pathway”, and other immune-related pathways. To further analyze the relationship between these targets and immunity, we enriched these targets for immune-related biological processes. The enrichment analysis result (Supplementary Figure S2B) showed that these targets are related to a variety of immune cells, such as T cells, macrophages, and DCs. Consistently, the effects of PG-related ingredients on the activation of macrophages and the activation and maturation of DCs have been reported (Wang et al., 2004; Park et al., 2014). These data have demonstrated the reliability of the prediction method and have further proved that PG has great potential in the treatment of cancer and immunomodulation.

Finally, to investigate whether these compounds have the potential to enhance patient survival, we tested the association of their targets with the clinical outcome in LUAD. For this, we applied approaches such as PLAGE (Pathway Level Analysis of Gene Expression) (Tomfohr et al., 2005), ssGSEA (single sample GSEA), GSVA (Gene Set Variation Analysis) (Hänzelmann et al., 2013), and Z-score gene set enrichment (GSE) analysis. These are robust and flexible GSE methodologies to model the overall expression level within highly heterogeneous gene expression profiles. The Kaplan–Meier curves (Figure 1C) showed that the targets of PG were closely related to LUAD survival. We further analyzed 175 targets of PG with patients survival and tumor stages in LUAD and found that the targets of PG were closely related to LUAD survival and tumor stages (Figure 1D; Supplementary Figure S2C). Taken together, these prediction results indicate to the great potential of PG in resisting tumor growth.

### *Platycodon grandiflorum* Inhibits Tumor Growth *In Vivo* and *In Vitro*

**FIGURE 2:**
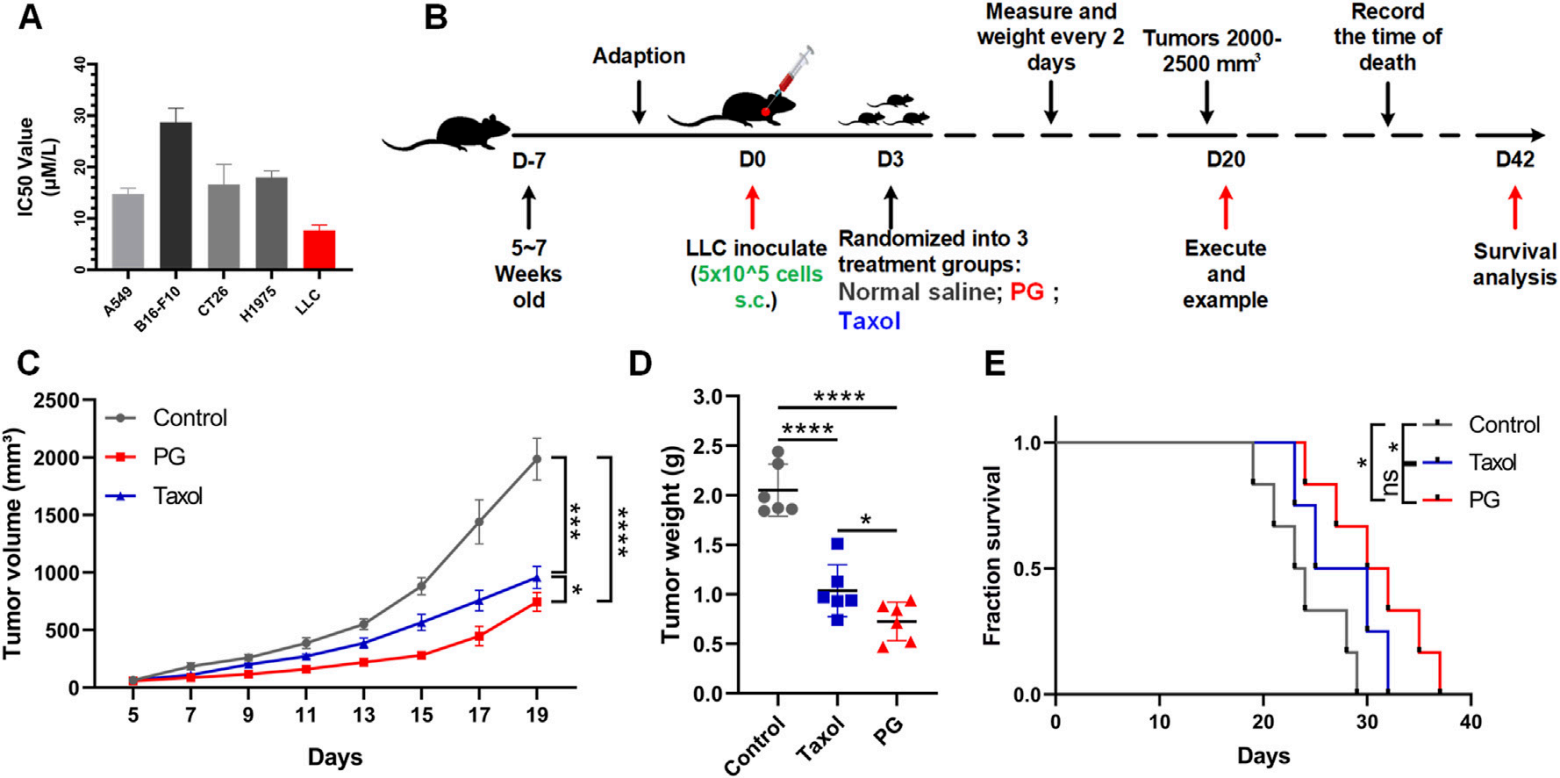
PG inhibits tumor growth *in vivo* and *in vitro*. **(A)** The cell viabilities of multiple tumor cell lines (A549, B16-F10, CT26, H1975, and LLC) treated with PG (5–50 μM). PG or control treatment was initiated when the cells reached the logarithmic growth stage and was determined after 48 h of incubation with PG (5–50 μM). Data are representative of three independent experiments. The results are the means ± SD of three independent experiments. **(B)** Schema of sample collection and analysis for LLC tumor-bearing mice. **(C)** Tumor volume changes in mice treated with PG (10 mg/kg), normal saline (model), or Taxol (15 mg/kg) are shown. **(D)** Tumor weight of sacrificed mice on day 20. PG vs. control. **(E)** Survival analysis for the mice. Survival in PG group was significantly better than the control group. The results shown (mean ± SD) reflect five or six mice/group, *p < 0.05, **p < 0.01, ***p < 0.001, and ****p < 0.0001.

Next, we investigated whether PG could inhibit the proliferation and viability of tumor cells and tumor growth in a xenograft model. *In vitro*, we used PG (PD: PD3 = 1.5:1) to treat multi-type cell lines: LLC, H1975, A549, CT26, and B16-F10. Increasing concentrations of PD reduced cell viability of the five cell lines after 48 h of treatment, with IC50 values ranging between 6.634 and 28.33 μM (Supplementary Figure S3A). Among them, LLC was the most sensitive to PD treatment (Figure 2A). The result verified that PG possesses a broad-spectrum of inhibitory activities on tumor cells.

*In vivo*, LLC-bearing C57BL/6 mice were constructed preferentially so as to investigate the *in vivo* therapeutic impact of PG on the growth of lung cancer (Figure 2B). Taxol, an effective chemotherapy drug for cancer treatment, was used as the positive control. As expected, according to the tumor volume changes shown in Figure 2C, we could recognize that the tumor volume in the control group increased rapidly (from 1,765 to 2,207 mm^3^), while the tumor volume of PG (from 668 to 891 mm^3^) and Taxol (from 821 to 1,050 mm^3^) grew slowly. On the 19th day, PG (744 mm^3^, *p* < 0.0001) significantly inhibited the growth of xenografted LLC tumors by 62.38% when compared with the control group (1986 mm^3^), which was slightly higher than Taxol (51.50%, 956 mm^3^, and *p* = 0.0001) (Supplementary Figure S3B). Likewise, the tumor weight analysis (Figure 2D) showed that the mean tumor weight of the PG group (0.73 g, *p* < 0.0001) and the Taxol group (1.03 g, *p* < 0.0001) was markedly lower than that of the control group (2.05 g). Another robust evidence for these findings was provided through survival analysis: mice treated with PG showed a statistically significant extension when compared with the control (day 29) which were extended to day 37 (Figure 2E). The *in vivo* results indicated that PG could slow down the process of tumor development obviously. Collectively, these results show that PG does have the ability to inhibit tumor growth and prolong survival.

### *Platycodon grandiflorum* Enhances Antitumor Immunity *via* Reducing Programmed Death-1 on the Surface of CD8^+^ T Cells

**FIGURE 3:**
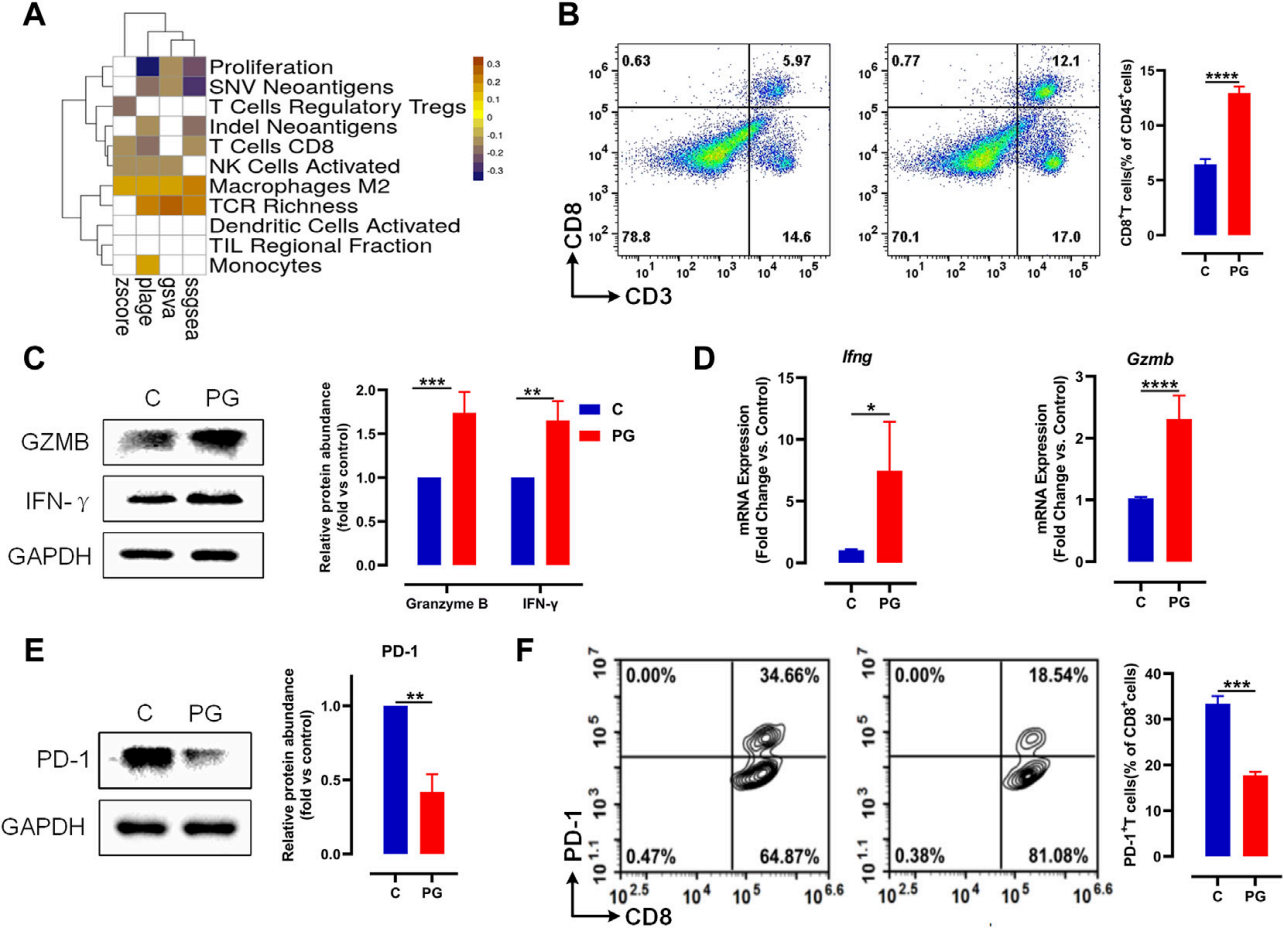
PG enhances antitumor immunity *via* reducing PD-1 on the surface of CD8^+^ T. **(A)** The heat map of Pearson correlation coefficients (PCCs) evaluated by the four methods between the gene expression level of PG targets and immune phenotypes; Benjamini–Hochberg (BH) adjusted *p*-values of PCCs <0.05 are shown in white. **(B)** LLC tumor infiltrating CD8^+^ T cells in PG-treated versus WT mice analyzed by flow cytometry. Flow cytometry analysis of single-cell suspensions obtained from LLC tumors and stained for detecting CD8^+^ T cells (CD3+CD8+). Representative dot plots with indicated percentages of CD8^+^ T cells populations within CD45^+^ cells in LLC tumors from the WT and PG-treated groups, respectively. **(C)** Protein level of IFN-γ and granzyme B in WT and PG-treated groups by means of western blotting. **(D)** Transcription levels of IFN-γ and granzyme B in WT and PG-treated groups by means of RT-PCR. **(E)** Protein level of PD-1 in WT and PG-treated groups. **(F)** Flow cytometry analysis of single-cell suspensions obtained from LLC tumors and stained for detecting PD-1^+^ CD8^+^ T cells. Representative dot plots with indicated percentages of PD-1^+^ CD8^+^ T-cell populations within CD8^+^ T cells in LLC tumors from WT and PG-treated groups, respectively. All plots of flow cytometry analysis were gated on total live cells. Data are from distinct samples and presented as the mean ± SEM. **p < 0.01, ***p < 0.001 compared with WT by unpaired t-test; n > = 3.

We have proved that PG possesses positive interference during tumor growth. To explore the exact mechanism of PG inhibition on tumor, we used PLAGE, ssGSEA, GSVA, and Z-score gene set enrichment analysis approaches to analyze the correlation between PG and the immunophenotype. The result (Figure 3A) showed that most of the immunophenotypes associated with the PG targets are related to T cells, for example, “T cells Regulatory Tregs”, “T cells CD8”, “TCR Richness”, and “TIL Regional Fraction”. The correlation analysis of each target and immunophenotype also proves this point of view (Supplementary Figure S4A). Therefore, we prioritized detecting the infiltration of CD8^+^ T cells, the most essential tumor killer cell in the tumor microenvironment, in fresh tumor samples following the flow cytometry analysis strategy in Supplementary Figure S4B. As shown in Figure 3B, the infiltration ratio of the CD8^+^ T cells after PG treatment was significantly higher than that of the normal saline–operated counterparts (Figure 3B).

We further tested the expression of interferon (IFN)-γ and granzyme B, which are primary cytokines for CD8^+^ T cells to kill tumors in TME (St Paul and Ohashi, 2020). Compared to the control group, PG upregulated the expression of them (Figures 3C,D), demonstrating that PG enables to increase the ability of killing tumor for CD8^+^ T cells. We next sought to determine how PG results in the killing activity of CD8^+^ T cells. Interestingly, we observed that PG reduces the expression of PD-1, a type of immune checkpoint protein expressed on immune cells, in the tumor samples (Figure 3E). The reduction of PD-1 indirectly explains that CD8^+^ T cells maintain the production of high levels of cytokines (McLane et al., 2019). To confirm PG increases CD8^+^ T cells viability through PD-1, we detected the number of PD-1^+^ CD8^+^ T cells in the TME. In sharp contrast, PG-treated mice (18.54%) showed lower number of PD-1^+^ CD8^+^ T cells infiltration than did the control (34.56%) (Figure 3F). Together, these results indicate that PG reduces PD-1 expression of CD8^+^ T cells in the TME.

### *Platycodon grandiflorum* Decreases Programmed Death-1 Through VEGF-A–VEGFR-2 Axis

**FIGURE 4:**
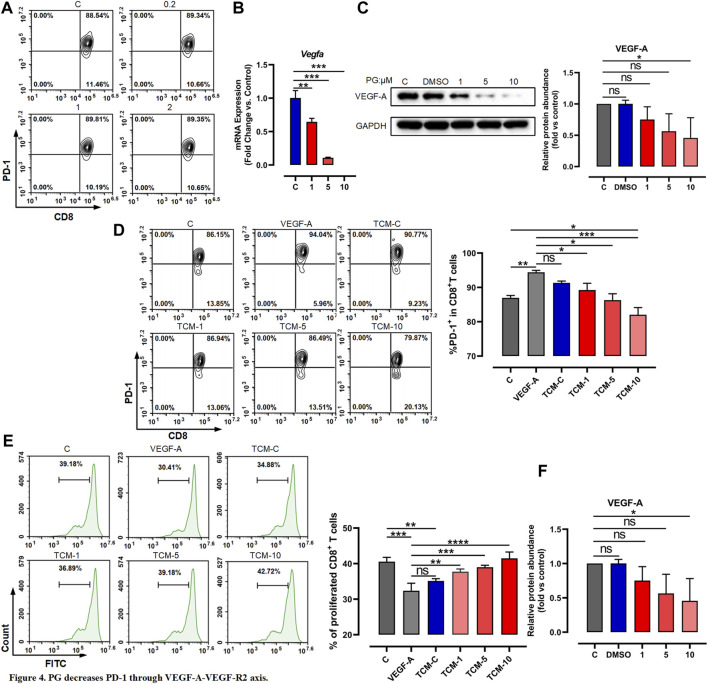
PG decreases PD-1 through VEGF-A–VEGFR-2 axis. **(A)** Flow cytometer detection of PD-1 expression in CD8^+^ T cells after treatment with PG. **(B)** Changes of VEGF-A transcription level in LLC after PG treatment. **(C)** Western blot analysis of the effect of PG on the expression of VEGF-A protein. **(D)** The proportion of PD-1^+^ CD8^+^ T cells in CD8^+^ T cells was tested by flow cytometry. Treatment of CD8^+^ T cells includes different tumor-conditioned media (TCM-C, TCM-1, TCM-5, and TCM-10) groups, and the blank control group and VEGF-A group. The % PD-1^+^ CD8^+^ T cells were quantified and are presented in the right panel. **(E)** The ratio of CD8^+^ T cells proliferation under different treatments: tumor-conditioned media (TCM-C), TCM-1, TCM-5, TCM-10, blank control group, and VEGF-A group. The % proliferative CD8^+^ T cells were quantified and are presented in the right panel. **(F)** Transcription levels of VEGFR-1 and VEGFR-2 were detected by RT-qPCR. Bar chart shows quantification of transcriptional levels compared to GAPDH control in each condition. The results are presented as the mean ± SD of triplicate determination. *p < 0.05, **p < 0.01, and ***p < 0.001.

To further investigate whether PG directly or indirectly promotes the lasting response of CD8^+^ T cells by downregulating PD-1, we first tested whether PG decreases the PD-1 expression of CD8^+^ T cells *in vitro*. We used low-dose PG (0, 0.2, 1, and 2 μM) to treat spleen cells activated for 3 days, and the flow cytometry results showed that PG has no obvious regulatory effect on the expression of PD-1 on the surface of CD8^+^ T cells (Figure 4A). The PD-1 protein level of the spleen cells after PG treatment was detected with similar results (Supplementary Figure S5A). We additionally tested the effect of PG on CD8^+^ T cells proliferation. Similarly, we did not observe PG promotes their proliferation (Supplementary Figure S5B, S5C). Overall, our data show that PG does not affect PD-1 expression and proliferation of CD8^+^ T cells directly.

As previous reports have described that among immunosuppressive factors produced by tumor cells, VEGF-A has a key role in the induction of an immunosuppressive microenvironment and can promote the expression of immunosuppressive factors such as PD-1 *via* the VEGF-A–VEGFR-2 axis (Gavalas et al., 2012; Costache et al., 2015; Voron et al., 2015; Yang et al., 2018). We therefore tested whether the activation of CD8^+^ T cells is indirectly caused by the reduction of VEGF-A derived from tumor cells mediated by PG. We first examined whether PG influences the expression of VEGF-A. To test this, we chose CT26 tumor cells known to produce high levels of VEGF-A *in vitro* (Terme et al., 2013)—similar evidence is provided in Supplementary Figure S5D—to test the impact of PG on VEGF-A. As shown in Supplementary Figures S5E and Figures 4B,C, PG can curb the production of VEGF-A in a dose-dependent manner, confirming that PG have the ability to inhibit the expression of VEGF-A of CT26 and LLC. We next used prepared tumor-conditioned medium (Supplementary Figure S5F) to culture CD8^+^ T cells so as to detect the influence of VEGF-A in the medium on the expression of PD-1 of CD8^+^ T cells. Compared with the conditioned medium treatment group alone, we observed that the PG-treated tumor-conditioned medium (PG-TCM) significantly decreased the number of PD-1^+^ CD8^+^ T cells (Figure 4D). In agreement with these results, PG-TCM also promoted proliferation of CD8^+^ T cells (Figure 4E). Furthermore, the result of the transcriptional level of VEGF-R1 and VEGFR-2 indicated that PG does indeed reduce the expression of VEGFR-2 by controlling VEGF-A (Figure 4F). In sum, our results implicate that PG indirectly reduces PD-1 on CD8^+^ T cells through the VEGF-A–VEGFR-2 axis.

### *Platycodon grandiflorum* Downregulates VEGF-A by p-STAT3 Signal

Given the above findings, we next explored how PG affects the reduction of VEGF-A. A large amount of research data show that VEGF-A is a direct target of STAT3, and the excessive activation of STAT3 causes the malignant development of tumors and is related to the poor prognosis of patients (Niu et al., 2002; Wei et al., 2003; Yu and Jove, 2004; Al Zaid Siddiquee and Turkson, 2008; Yu et al., 2009; Johnston and Grandis, 2011; Dimri and SukanyaDe, 2017; Gao et al., 2017; Huynh et al., 2019). Based on these, we surmised that the decrease of VEGF-A may be mediated by the over-activation of PG-regulated STAT3.

**FIGURE 5:**
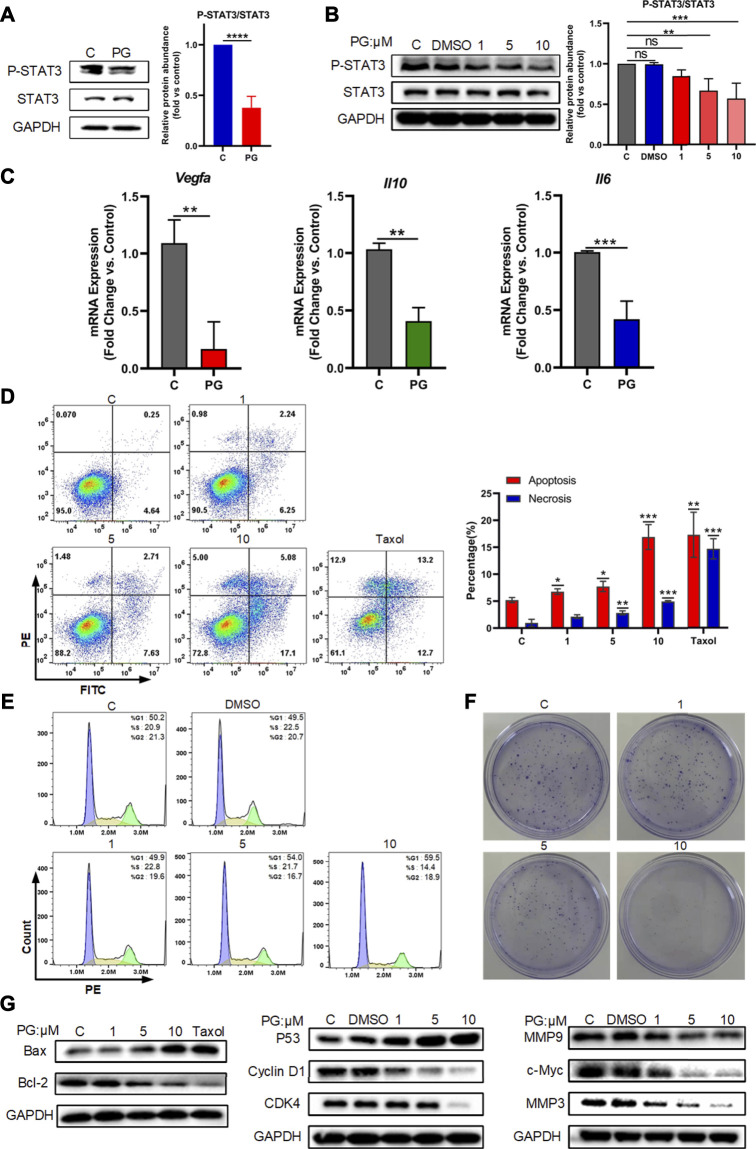
PG downregulates VEGF-A by p-STAT3 signal. **(A)** Protein levels of STAT3 and P-STAT3 in WT and PG-treated groups. **(B)** Protein expressions of STAT3 and P-STAT3 after treated by PG were tested by Western Blot analysis. Bar chart shows quantification of protein levels compared to GAPDH control in each condition. **(C)** The transcriptional level of the key downstream factors of STAT3: IL-10, IL-6, and VEGF-A in tissues of control and PG-treated groups. **(D)** Apoptosis-inducing effect of PD on LLC cells. Annexin V/propidium iodide (PI) staining using flow cytometry was performed after LLC cells were treated with various concentrations of PG for 48 h. Taxol at concentration of 250 nM was used as positive control and DMSO (0.05%) without PG was used as negative control. Apoptosis and necrosis were quantified in the right histogram as the percentage of apoptosis. Quantitative analysis of the results of flow cytometry analysis presented the mean ± standard deviation of triplicate. **(E)** Cell cycle distribution was assayed with PI staining by flow cytometry after 24 h of treatment with or without PG. Percentage of cells in G1, S, or G2 phases of the cell cycle presented in the right bar chart. Data are presented as the mean ± standard deviation of triplicates. **(F)** Effect of different concentrations of PG on the proliferation in LLC cells was measured by a colony formation assay. **(G)** The expression of apoptosis-related proteins, cell cycle‐related proteins and cell cycle‐related proteins in LLC cells treated with or without PG and Taxol was estimated by western blot analysis after 48 h (From left to right). All quantitative data are presented as the mean ± standard deviation of the mean. *p < 0.05, **p < 0.01, and ***p < 0.001.

Considering that phosphorylation of Y705 of STAT3, a major STAT3 phosphorylation way, is involved in tumorigenesis (Dimri and SukanyaDe, 2017), we accordingly tested whether PG would affect the Y705 phosphorylation level of STAT3. We observed that PG reduces the production of STAT3 phosphorylation at the tissue and cell level (Figures 5A,B). Corroborative evidence for reduction of STAT3 phosphorylation also comes from the detection of the other two key factors, IL-6 and IL-10, of STAT3 (Yu et al., 2007), and for which PG also downregulated the expression of them (Figure 5C). We indeed found that the PG significantly suppresses the production of VEGF-A through the inhibition of activation of the STAT3 signal.

Activation of STAT3 is considered to be involved in the regulation of a variety of critical functions, namely, cell proliferation, cell cycle, apoptosis, etc. (Yu et al., 2007). We also tried to detect the effects of PG on the occurrence of the three biological processes of apoptosis, cell cycle, and proliferation mediated by P-STAT3 at the cellular level. For this, we first tested the effects of different concentrations of PG on the apoptosis rate of tumor cells through flow cytometry analysis, and we found PG promotes tumor cell apoptosis is concentration dependent (Figure 5D). At the highest treatment concentration of 10 μM, the apoptotic rate increased to 3.68 times that of the vehicle group, which was slightly higher than that of the positive group (2.74 times). Corroborating evidence was provided through detecting the expression level of apoptosis-related proteins (Figure 5G, Supplementary Figure S6C). We then used the same method to detect the effect of PG on the cell cycle. In sharp contrast, PG inhibits the cell cycle in the G1 phase in a concentration-dependent manner (Figure 5B, Supplementary Figure S6A). We got similar results in the detection of cell cycle–related proteins (Figure 5G, Supplementary Figure S6B). We finally utilized the colony formation assay which would reflect the degree of proliferation by observing the number of cell colonies. Strikingly, the number of cell clones decreased with increasing PG concentration (Figure 5F), which was consistent with the results of proliferation-related protein testing (Figure 5G, Supplementary Figure S6D). Collectively, these results suggest that PG can mediate tumor cell growth by downregulating STAT3 phosphorylation.

### Combination of *Platycodon grandiflorum* and Anti–Programmed Death Ligand-1 Does Not Enhance the Antitumor Effect

**FIGURE 6:**
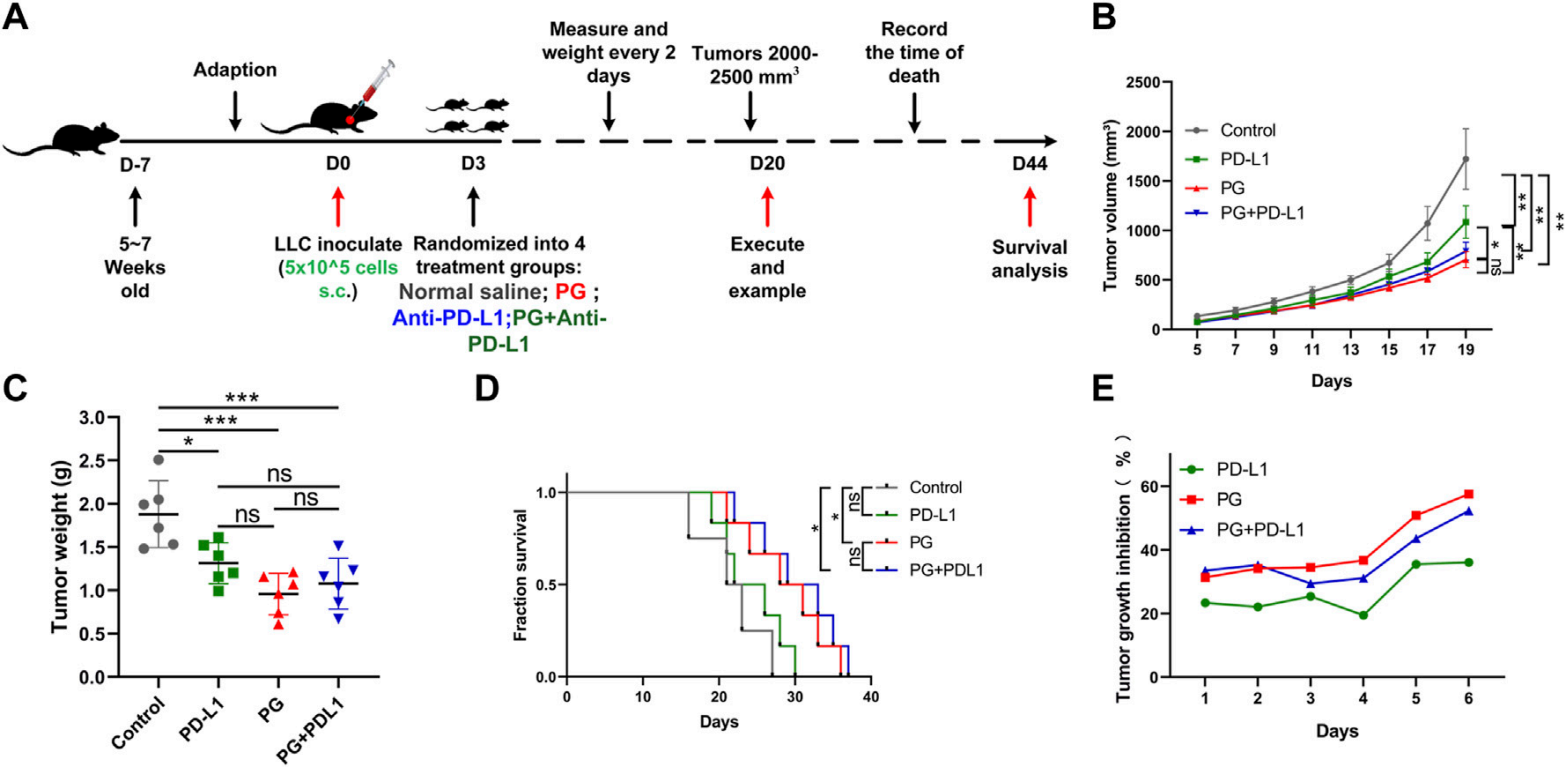
The combination PG and anti–PD-L1 do not enhance the antitumor effect. **(A)** Schema of sample collection and analysis for LLC tumor-bearing mice. **(B)** Tumor volume changes in mice treated with PG (10 mg/kg), anti–PD-L1 (200 µg per mouse), combination of PG and anti–PD-L1, and saline (model) are shown. **(C)** Tumor weight of sacrificed mice on day 20. **(D)** Survival analysis for the mice. **(E)** Tumor growth inhibition rate (TGI) of the mice. The results shown (mean ± SD) reflect five or six mice/group, *p < 0.05, **p < 0.01, ***p < 0.001, and ****p < 0.0001.

Having verified the exact mechanism of PG-mediated antitumor effects in the TME through studying, and to determine whether the combination of PG and anti–PD-L1 antibody enhances the therapeutic effect of antitumor, an animal experiment was performed as shown in Figure 6A. Compared with the control (Figure 6B), PG and anti–PD-L1 suppressed tumor growth by 57% and 36%, respectively, whereas the combination of PG and anti–PD-L1 group (CP) reduced tumor growth by 52% at day 18 (Figure 6E). No significant difference in tumor volume was observed between the PG and the CP groups, showing that CP does not exert a better curative effect when compared with PG alone. Moreover, the tumor weight differences analysis found that PG could slow down tumor growth evidently as CP (Figure 6C). The results of survival analysis once again proved that PG has a strong tumor treatment effect; however, the CP group did not show better ability to prolong survival (Figure 6D). Obviously, these data suggest that CP is not sufficient to significantly gain better antitumor effects.

In sum, our work shows that PG, a widely used traditional Chinese medicine, not only reduces the infiltration of PD-1^+^ CD8 T^+^ cells by regulating the VEGF-A–VEGFR-2 axis to enhance the killing activity of CD8 T^+^ cells but also decreases the production of the phosphorylation of STAT3 of tumor cells to mediate the development of tumor growth in the TME. Through the combination of these two effects, PG can exert a powerful therapeutic antitumor effect.

## Discussion

Immune checkpoint therapies (ICT), especially monoclonal antibodies targeting the PD-1/PD-L1 immunosuppressive pathway to release the CD8^+^ T-cell killing activity, have made breakthrough progress clinically. The emergence of these brings great hope because of their improving durable outcomes for cancer patients. However, accumulating evidence suggests that severe irAEs are seen in some patients undergoing ICI therapy (Boutros et al., 2016; Wang et al., 2019; Ramos-Casals et al., 2020). The fundamental reason is inhibition of immune checkpoints reinforcing the normal physiological barriers against autoimmunity, leading to various local and systemic autoimmune responses. Therefore, the development of inhibiting the local PD-1/PD-L1 signaling pathway of the TME is critical to avoid any adverse effects. Ongoing clinical studies are aiming to develop novel immunotherapy drugs for better treatment outcomes and less irAEs. Herein, we found *P. grandiflorum* reduces the expression of PD-1 on the surface of CD8^+^ T cells to exert antitumor effects in NSCLC. The data demonstrate that PG may mediate antitumor immunity through immune regulation of CD8 T^+^ cells in the TME.

Since the expression of PD-1 on the surface of CD8 T^+^ will limit the killing activity of CD8 T^+^ cells, we hypothesized that PD-1 downregulation mediated by PG results in intratumoral CD8 T^+^ cells infiltration and associated changes in the cytokine. Indeed, in PG-treated tumors, we observed that the infiltration of PD-1^+^ CD8 T^+^ cells was decreased and the production of the killing factors: IFN-γ and Granzyme B were increased. To explore the reasons for PD-1 downregulation, we noticed PG reduces the production of inhibitory factor VEGF-A which regulates the expression of PD-1 on the surface of CD8 T^+^ cells *via* VEGF-A–VEGFR-2 (Gavalas et al., 2012; Costache et al., 2015; Voron et al., 2015). We cultured activated CD8^+^ T cells in conditional medium *in vitro* and found that PG indirectly regulates VEGF-A–VEGFR-2 by reducing the release of the inhibitory factor VEGF-A from the tumor cells to immunomodulate the expression of PD-1 on the surface of CD8 T^+^ cells, rather than directly acting on CD8^+^ T cells, and experimental verifications reveal that the downregulation of VEGF-A is due to the fact that PG reduces the phosphorylation of STAT3 in tumor cells, which additionally affects apoptosis, proliferation, and cycle biological processes mediated by STAT3 downstream (Yu et al., 2007). This study uncovered a new insight into how PG regulates CD8^+^ T-cell immunity to inhibit tumor growth in the TME. Compared with previous antitumor research on PG and its main components, our study answers whether PG inhibits tumor growth through immune regulation, and the mechanism(s) of how PG exerts the antitumor immunity. In this study, we have identified PG as a low-side-effects drug inhibiting the production of PD-1 at the surface of CD8 T^+^ cells against tumor growth through reducing the expression of VEGF-A derived from tumor cells. The emerging evidence suggests a multi-faceted immunomodulatory role for PG in regulating tumor immune microenvironment. It should be noted that a number of studies have shown that normal cells have high sensitivity to PD and PD3, which have no significant effects on normal cells (Jung et al., 2011; Chun et al., 2013; Ye et al., 2013; Lee et al., 2021), so we did not evaluate the cytotoxic activity on the normal cells in our cell experiments. Additionally, we found the intraperitoneal injection of PD and PD3 have a strong lethal effect through preliminary experiments in the early stage of the study: the weight of the mice was reduced, a burning mark appeared in their abdomen, and they died a few days later. This is as reported in many literature: it has a strong hemolytic activity (Sun et al., 2011; Li et al., 2015b; Tao et al., 2015; Li et al., 2016c), therefore, our method of administration of the combination of PD and PD3 is gavage instead of intraperitoneal injection.

The development of immunotherapy drugs those target CD8^+^ T cells in the TME and possess low side effects is an urgent task in current cancer therapy. Our research on PG shows PG might serve as an appropriate immunotherapy drug to enhance CD8^+^ T killing activity of local TME rather than as monoclonal antibodies to change the entire immune state of the immune system. Although our animal model data suggested favorable antitumor effects, many questions about how best to incorporate PG into immunotherapy strategies will need to be studied in prospective research work. In this study, we also tried to study whether PG combined with anti–PD-L1 antibody can enhance the inhibition of tumor growth. However, the combination did not improve the outcome of tumor treatment. We speculate that this may be due to the same signaling pathways mediated by the drug *P. grandiflorum* and anti–PD-L1 antibody. This also needs to be further demonstrated in future work. The mechanism of immunotherapy of PG in NSCLC provides an evident reference for treatment of cancer to prevent irAEs and further enhance antitumor efficacy.

## Materials and Methods

### Pharmacokinetic Screening

All the compounds of *P. grandiflorum* come from the Traditional Chinese Medicine Systems Pharmacology Database (TCMSP, http://tcmspw.com/) and manual literature mining. In order to obtain the potential active compounds, we used ADME parameters: OB ≥ 20%, DL ≥ 0.18, and HL = long to screen.

### Target Prediction

The potential targets for PG were predicted by utilizing the weighted ensemble similarity (WES) and systematic drug targeting tool (Sys DT) methods. For the supplement of protein information, refer to UniProt database (http://www.uniprot.org/). The compounds–corresponded targets information was obtained through the above steps.

### C-T Network

According to the relationship between compounds and targets, we constructed a C-T network by *Cytoscape 3.7.1*. Among them, the V type represents the active compound, the circle represents the target, edge, and connection of the lines representing the relationship between them.

### High-Performance Liquid Chromatography

High-performance liquid chromatography (HPLC) analysis method was used to identify the main compound of the extract of PG.

### GOBP (Gene ontology-biological process) and KEGG (Kyoto Encyclopedia of Genes and Genomes) Analysis

To understand the distribution of targets in the pathways and biological processes, we performed the GO enrichment analysis and KEGG pathway enrichment analysis by linking the targets to ClueGO (Cytoscape plugin). Only *p*-values ≤ 0.05 were selected for displaying in the Enrichment Map.

### Analysis of the Correlation Between the Targets and Lung Adenocarcinoma Patient

We analyzed the correlation between potential targets and lung adenocarcinoma (LUAD) clinical features, disease stage, and overall survival time. The RNA-seq data and relative information of LUAD in the TCGA were download by the R TCGAbiolinks package. Among them, LUAD clinical features mostly related to immunity, such as regulatory T cells, CD8 T cells, NK cells, M2 macrophages, DCs, TIL regional fraction, monocytes, and TCR richness. The correlation of the mRNA level [log2 (TMP+1)] of targets with these phenotypes was analyzed by Pearson’s correlation coefficient (PCC). The *p*-values of PCCs were adjusted by the Benjamini–Hochberg methods. PLAGE (pathway level analysis of gene expression), ssGSEA (single sample GSEA), GSVA (gene set variation analysis), and Z-score gene set enrichment (GSE) analysis approaches were used to evaluate the correlation between the targets and the patients' survival. Kaplan–Meier plots summarized the correlation between the proteins' expression levels and patient survival. In the correlation analysis of the 175 targets of PG with patient survival and tumor stages in LUAD, Z values and T values were obtained from the Cox regression model and linear regression, respectively.

### Mouse Tumor Model

Female C57BL/6 mice (5 weeks, 18–22 g) used in this study were obtained from the Comparative Medicine Centre of Yangzhou University (Yangzhou, China). They were bred and maintained in the Institute of Laboratory Animals of Pharmacology Toxicology of Jiangsu Kang yuan Pharmaceutical Co., Ltd., where they were provided a special pathogen-free environment. The mice were housed socially (six mice per cage) on a 12-h light/dark cycle in individually ventilated cages, with *ad libitum* access to food and water. The experimental holding room had a temperature of 25 ± 1°C and humidity control of 55 ± 5% and was supplied with High Efficiency Particle Air (HEPA)-filtered air. All animal experiments were performed in accordance with the national and institutional guidelines for animal care.

After adoption for 1 week, the LLC cells, resuspended in 100 µl PBS (5 × 10^5^ cells/mouse) were transplanted subcutaneously into the left axillary region of each mouse and were allowed 3 days to establish tumors. Subsequently, the mice were randomly assigned to 10 experimental groups: 2 groups of control (*n* = 6/group, intraperitoneal injection of physiological saline); 2 groups of positive control (*n* = 6/group, daily intraperitoneal injection of Taxol, 15 mg/kg, YuanYe, Shanghai); 2 groups of PG (*n* = 6/group, daily intragastric administration of PG, 10 mg/kg, YuanYe, Shanghai); 2 groups of anti–PD-L1 (clone B7-H1) (BE0101, BioXCell) (*n* = 6/group, intraperitoneally injection on days 4, 7, and 10, 200 µg per mouse); 2 groups of the combination of PG and anti–PD-L1 (*n* = 6/group, the method of administration is as above). Half of them were used for the analysis of tumor volume and weight. When the tumor reached a size of 50 mm^3^, the administration was started immediately and the tumor size measured every other day until the tumor was larger than 2,500 mm^3^, then the mice were sacrificed by cervical dislocation which was recorded as death. Meanwhile, the tumor was taken out for weight analysis. The tumor volume was estimated as follows: length × width × 0.5 (Yamazaki et al., 2002). While the other half was used for survival analysis. Moreover, the tumor growth inhibition rate (TGI, %) was calculated as follows: TGI (%) = [1 − (tumor volume in the treated group)/(tumor volume in the control group)] × 100.

### Cell Lines and Cultures

LLC (mouse lung cancer cell line), H1975 (human lung cancer cell line), A549 (human lung cancer cell line), CT26 (mouse colon cancer cell line), and B16-F10 (mouse melanoma cell line) were obtained from the Cell Bank of the Chinese Academy of Sciences, Shanghai. Among these, LLC and A549 were maintained in the DMEM, while the remaining were maintained in RPMI-1640. These cell lines were cultured at 37°C in a 5% CO_2_ atmosphere. To ensure cell viability, the culture medium was refreshed every 2–3 days, passaging or harvesting when the cells reached approximately 80–90% confluency. All media used in this study were supplemented with 100 U/ml penicillin and 100 mg/ml streptomycin. RPMI-1640 and DMEM were also supplemented with 10% fetal bovine serum (FBS) (10099141, Gibco, Thermo Fisher Scientific).

### Cell Viability Assays

The cytotoxicity and ability of cell proliferation were evaluated by the Cell Counting Kit-8 (CPG-8). LLC, H1975, A549, CT26, and B16-F10 cell lines in the logarithmic phase, which were seeded in 96-well plates at the density of 1 × 10^5^/ml or 8 × 10^4^/ml and volume of 100 μl and had completely adhered to the wall 24 h later. Then, the cells were treated with various concentrations of PG (50, 40, 25, 20, 12.5, 10, 6.25, 5, and 0 μM) maintained for 48 h. Subsequently, the original medium was removed and refreshed with 100 μl CCK-8 solution prepared according to 10 (culture medium): 1 (CCK-8) for 2–4 h incubation under 37°C and 5% CO_2_ conditions. Finally, the absorbance was detected by a microplate reader (Molecular Devices, California, United States) at 450 nm. A total of 4–6 reduplicate wells were used for each treatment, and the evaluation of different cells were performed three times. The half maximal inhibitory concentration (IC50, %) was calculated as follows: cell viability = (OD_Drug_ − OD_Blank_)/(OD_Control_ − OD_Blank_) × 100%, and the results of IC50 were visualized *via* GraphPad Prism 8.0.2.

### Western Blotting

Detecting changes in the protein level by Western blotting. The tissue samples were lysed in RIPA Lysis Buffer (P0013B, Beyotime) mixed with phenylmethanesulfonyl fluoride (PMSF, ST506, and Beyotime) on ice, and the lysates were centrifuged at 4°C and 14,000 rpm for 10 min and collected as tissue–protein samples. The cell samples treated in the different experimental designs were scraped, collected by centrifugation, and lysed in the Qproteome™ Mammalian Protein Prep Kit (37,901, Qiagen). Subsequently, these samples were determined using the BCA Protein Assay Kit (P0012, Beyotime) and absorbance measurements were taken at 562 nm to evaluate the protein concentration. Quantified protein samples were mixed with loading buffer containing 5% β-mercaptoethanol and then denatured at 100°C for 10 min.

Equalized amounts of total protein from the samples were separated by SDS-PAGE and transferred to PVDF membranes (1620177, Bio-Rad). The membranes were blocked with 5% skim milk in TBST buffer for 2 h and were incubated with primary antibodies overnight at 4°C.

After washing, the membranes were incubated with secondary antibodies (Goat Anti-Rabbit IgG H&L, ab6721, Abcam) for 1 h at room temperature. Finally, the protein expression was detected and visualized using the enhanced chemiluminescent procedure (Clarity™ Western ECL Substrate, Bio-Rad, 170-5061), followed by scanning in a ChemiDoc™ XRS+ Imaging System (Bio-Rad). The primary antibodies used in this experiment are as follows: Anti-STAT3 antibody (ab68153, Abcam), Anti–P-STAT3 antibody (ab76315, Abcam), Anti–VEGF-A antibody (ab214424, Abcam), Anti-CDK4 (ab108357, Abcam), Anti-Cyclin D1 (ab134175, Abcam), Anti–BCL-2 antibody (sc-7382, Santa Cruz), Anti–c-Myc antibody (13987s, Cell Signaling Technology), Anti-BAX antibody (sc-526, Santa Cruz), Anti–MMP-3 antibody (ab52915, Abcam), Anti–MMP-9 antibody (sc-10739, Santa Cruz Biotechnology), Anti-p53 antibody (9282s, Cell Signaling Technology), Anti-GAPDH antibody (ab181602, Abcam), Anti–β-actin (ab8227, Abcam), Anti–Granzyme B antibody (ab208586, Abcam), Anti–IFN-γ antibody (ab133566, Abcam), and Anti–PD-1 antibody (ab214421, Abcam). ImageJ was used to relatively (no absolute values) quantify protein bands from western blot films. The quantification reflects the relative amounts as a ratio of each protein band relative to the control, and quantitative results were visualized through GraphPad Prism 8.0.2.

### Harvest Tissue and Prepare a Single-Cell Suspension

Fresh tumor tissues or spleens were mechanically dissociated to minced meat and digested for 30 min at 37°C in RPMI 1640 with 2 mg/ml Collagenase IV (17104-019, Gibco), 2000 U/ml DNase I (D7073, Beyotime Biotechnology), 0.5 mg/ml Hyaluronidase (S10060, YuanYe Biotechnology) and 0.5 mg/ml Dispase II (S25046, YuanYe Biotechnology). 20–30 ml of 1 × PBS containing 2% FBS (F-PBS) was added to stop the digestive reaction. Subsequently, red blood cell lysis was used to remove red blood cells for 5 min, and the lysis reaction then stopped by adding 20–30 ml of F-PBS. Then, the cell suspension was filtrated on a 70-μm cell strainer (15-1070, BIOLOGIX) and 40-μm cell strainer (15-1040, BIOLOGIX), and washed with F-PBS. In this way, a single cell suspension of the tumor tissue or spleen was obtained. Finally, the cells were counted and tested for viability by Countess^TM^ automated cell counter (C10281, Invitrogen) to wait for the next step.

### Intratumoral CD8 T^+^ cells or PD-1^+^ CD8^+^ T Detection

For analysis of the infiltrating cell number and the ratio of CD8 T^+^ cells and PD-1^+^ CD8 T^+^ cells, tumor-bearing mice were sacrificed and tumors were harvested after 18 days of drug treatment. Single-cell suspensions were prepared and incubated with Anti-Mouse CD16/32 purified (08212-20, Biogems) for 30 min at room temperature. Cells were then washed and stained with antibodies for surface markers for 30 min at room temperature. The fluorescent conjugated antibodies used are anti-mouse CD45 PE/Cy7 (103114, BioLegend), Anti-Mouse CD3e APC (05122-80-25, Biogems, PeproTech), anti-mouse CD8a PE (100707, Biolegend), and anti-mouse PD-1 FITC (135213, Biolegend). After washing in PBS, the cells were resuspended in F-PBS and analyzed with a NovoCyte Flow Cytometer (ACEA Biosciences) using FlowJo software (BD bioscience).

### CD8 T^+^ Cells Proliferation Assay and Programmed Death-1 Expression Detection *in vitro*

The untouched and highly purified mouse CD8^+^ T cells were isolated from single-cell suspensions of splenocytes by immunomagnetic negative selection using the EasySep Mouse CD8^+^ T-Cell Isolation Kit (19853, STEMCELL), according to the manufacturer’s instructions, and the CD8^+^ T cells purity (>90%) was identified by flow cytometry Anti-mouse CD3e APC (05122-80-25, Biogems, PeproTech) and anti-mouse CD8a PE (100707, Biolegend). Then, the CD8^+^ T cells were labeled with 1× CFSE (C0051, Beyotime) and were cultured at a density of 3 × 10^5^/ml into 96-well plate–bound 5 μg/ml CD3 overnight (05112-25-500, Biogems), 2 μg/ml CD28 (16-0281-86, eBioscience) and 30 U/ml IL-2 (78081, STEMCELL) were supplied to maintain their growth subsequently. To evaluate the direct effect of PG on T-cell proliferation directly *in vitro*, the activated CD8^+^ T cells were incubated in the presence or absence of low concentration of PG (0.2, 1, and 2 μM). Indirectly, the activated CD8^+^ T cells were incubated in the presence or absence of different tumor-conditioned media or positive control treated with 100 ng/ml VEGF-A (493-MV-005, R&D Systems). Tumor-conditioned media was manufactured as is shown in Supplementary Figure S4. Incubating under 37°C and 5% CO_2_ conditions for 3 days. Finally, the cells were harvested and analyzed by NovoCyte Flow Cytometer (ACEA Biosciences) using FlowJo software (BD Biosciences).

For PD-1 expression detection *in vitro*, the group design is the same as above. After culturing the cells to the specified number of days, the cells were collected and stained with anti-mouse CD8a PE and anti-mouse PD-1 FITC for 30 min. Washing the cells three times was performed with F-PBS and resuspended. The harvested cells were analyzed by NovoCyte Flow Cytometer using FlowJo software.

### Cell Cycle and Apoptosis Analysis by Flow Cytometry

LLC cells in the logarithmic growth phase were seeded onto a six-well culture plate at a density of 5 × 10^5^ cells per well and incubated at 37°C in a 5% CO_2_ humidified incubator in the presence of 0, 1, 5, and 10 μM PG or 250 nM Taxol as the positive control. After 48 h, apoptosis and necrosis were analyzed with the FITC Annexin V Apoptosis Detection Kit I (556547, BD Pharmingen) following the manufacturer’s instructions.

For cell cycle, LLC cells were seeded onto a six-well culture plate following the above method in the presence of 0, 1, 5, and 10 μM PG and DMSO (0.05%) as a negative control. Similarly, 48 h later, the effect on the cell cycle was detected with the Cell Cycle and Apoptosis Analysis Kit according to the manufacturer’s protocol. In these two methods, the cells were harvested and analyzed by NovoCyte Flow Cytometer using FlowJo software.

### Colony-Forming Assays

The colony formation assay was used to determine the self-renewal and proliferative capacity of tumor cells. LLC cells in the logarithmic phase were seeded in six-well plates at a density of 1000 cells/well in complete 1640 medium in standard culture conditions (5% CO_2_ and 37°C) for 24 h to allow them to attach to the plate. Then, treating them with various concentrations of PG: 0, 1, 5, and 10 (μM) and incubating the cells in a CO_2_ incubator at 37°C for 1–3 weeks until the cells in the control plates had formed colonies those were of a substantially good size (50 cells per colony is the minimum for scoring). Next, the colonies were fixed with 5 ml absolute methanol for 15 min at room temperature (RT), stained with 1% crystal violet for 30 min–1 h, washed with water three times to rinse off crystal violet, and air dried. The number of visible colonies was counted under a colony formation rate = (number of colonies formed/number of cells seeded) × 100%.

## RNA Isolation and Quantitative Real-Time Polymerase Chain Reaction

Total RNA was isolated using the RNeasy Mini Kit (74104, Qiagen, Germany) from tumor tissue or different treated cells, and cDNA was synthesized using the PrimeScript RT reagent Kit with gDNA Eraser (Perfect Real Time) (RR047A, Takara) according to the manufacturer’s manual. Quantitative real-time PCR (qRT-PCR) analysis was performed using TB Green® Premix Ex Taq™ II (TII RNaseH Plus) [RR820B (A × 2), Takara] according to the manufacturer’s instructions on a StepOnePlus^TM^ Real-Time PCR System (Applied Biosystems) using the relative standard curve method. The PCR conditions were 1 cycle at 95°C for 30 s, 40 cycles of 5 s at 95°C, and 34 s at 60°C. The data were analyzed by 2^−△△Ct^.

Primers for mIL-6 (mIL-6-forward 5′-AAG​TGC​ATC​ATC​GTT​GTT​CAT​ACA-3′ and mIL-6-reverse 5′-GAG​GAT​ACC​ACT​CCC​AAC​AGA​CC-3′), mIL-10 (mIL-10-forward 5′-TTT​TCA​CAG​GGG​AGA​AAT​CG-3′ and mIL-10-reverse 5′-CCA​AGC​CTT​ATC​GGA​AAT​GA-3′), mVEGF-A (mVEGF-A-forward 5′-AGT​ACA​TCT​TCA​AGC​CGT​C-3′ and mVEGF-A-reverse 5′-GCA​GGA​ACA​TTT​ACA​CGT​C-3′), mGranzyme B (mGranzyme B-forward 5′-TCA​GGC​TGC​TGA​TCC​TTG​ATC​G-3′ and mGranzyme B-reverse 5′-ATG​AAG​ATC​CTC​CTG​CTA​CTG​C-3′), mIFN-γ (mIFN-γ-forward 5′-AGG​AAC​TGG​CAA​AAG​GAT​GGT-3′ and mIFN-γ-reverse 5′-TCA​TTG​AAT​GCT​TGG​CGC​TG-3′), mVEGFR-1 (mVEGFR-1-forward 5′-CAG​GCC​CAG​TTT​CTG​CCA​TT-3′ and mVEGFR-1-reverse 5′-TTC​CAG​CTC​AGC​GTG​GTC​GTA-3′), mVEGFR-2 (mVEGFR-2-forward 5′-CCA​GCA​AAA​GCA​GGG​AGT​CTG​T-3′ and mVEGFR-2-reverse 5′-TGT​CTG​TGT​CAT​CGG​AGT​GAT​ATC​C-3′), mGapdh (mGapdh-forward 5′-TGA​CCT​CAA​CTA​CAT​GGT​CTA​CA-3′ and mGapdh-reverse 5′-CTT​CCC​ATT​CTC​GGC​CTT​G-3′).

## Data Availability

The data used to support the findings of this study are available from the corresponding author upon request.
