# Supplementary figures and images for: Platycodon grandiflorum Triggers Antitumor Immunity by Restricting PD-1 Expression of CD8+ T Cells in Local Tumor Microenvironment
Source: Front Pharmacol. 2022 Apr 14;13:774440. doi: 10.3389/fphar.2022.774440 (PMC9046572; doi:10.3389/fphar.2022.774440)

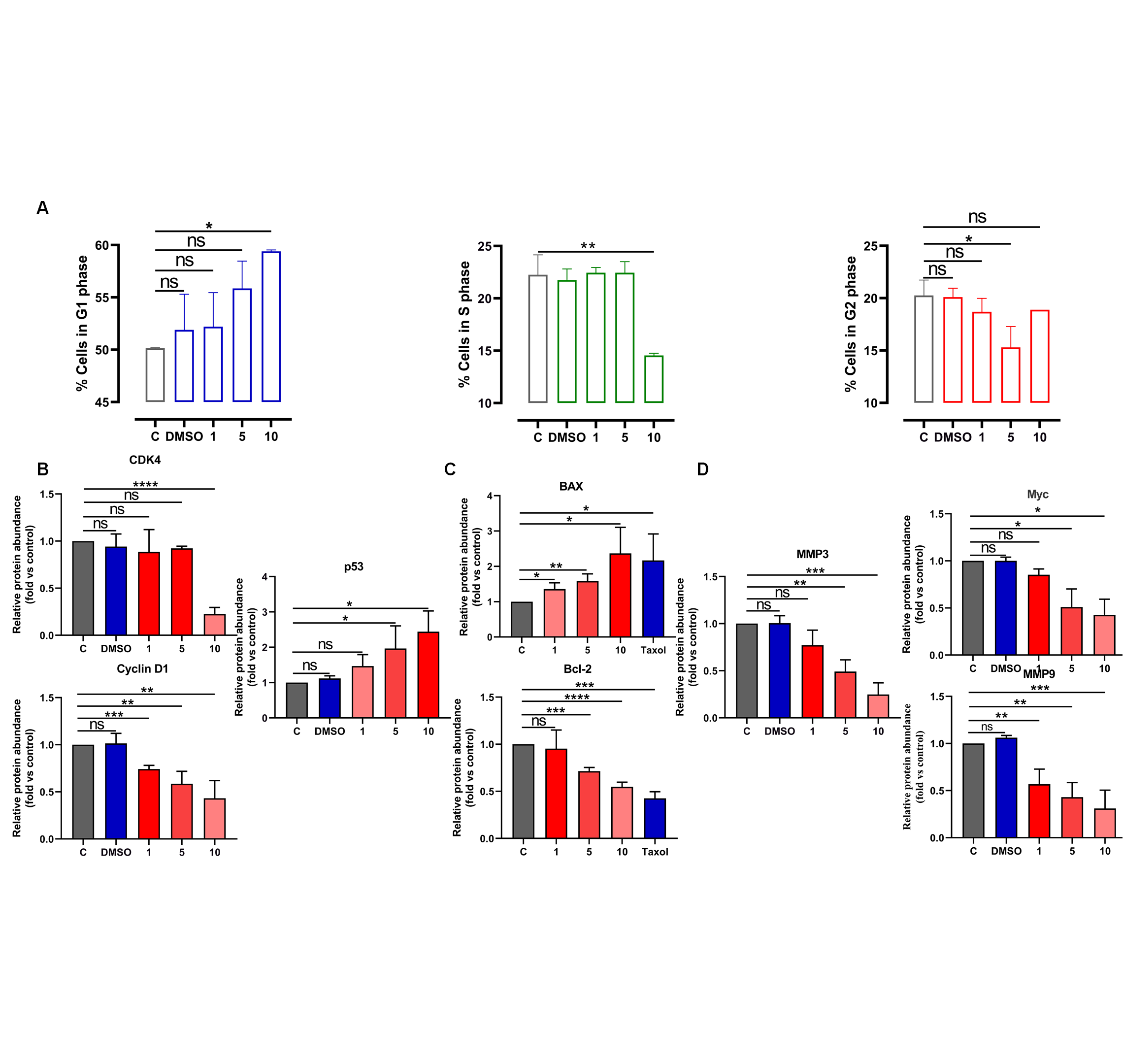

Supplement: Supplementary file 2 [file Image6.TIF]

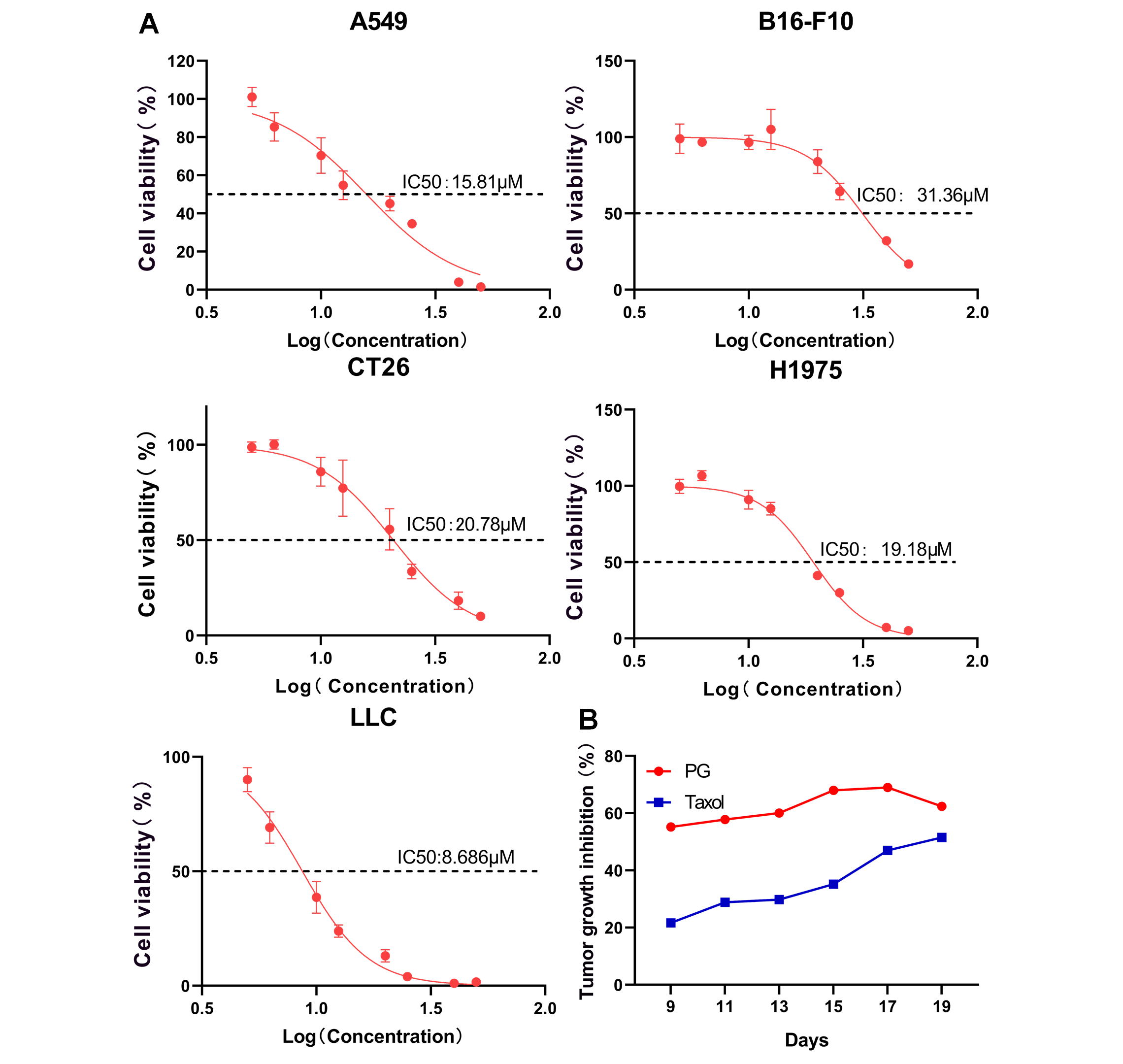

Supplement: Supplementary file 4 [file Image3.TIF]

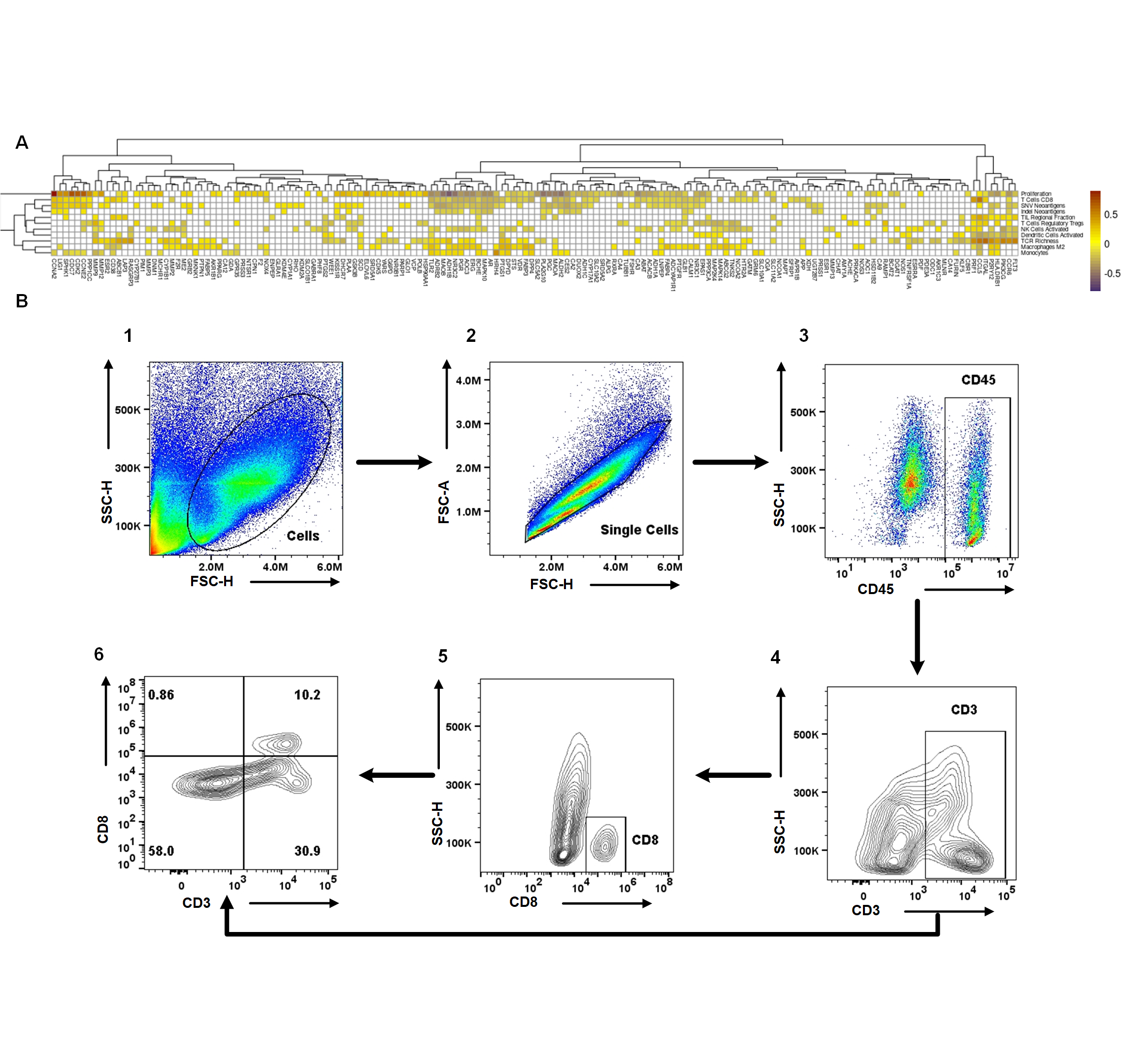

Supplement: Supplementary file 5 [file Image4.TIF]

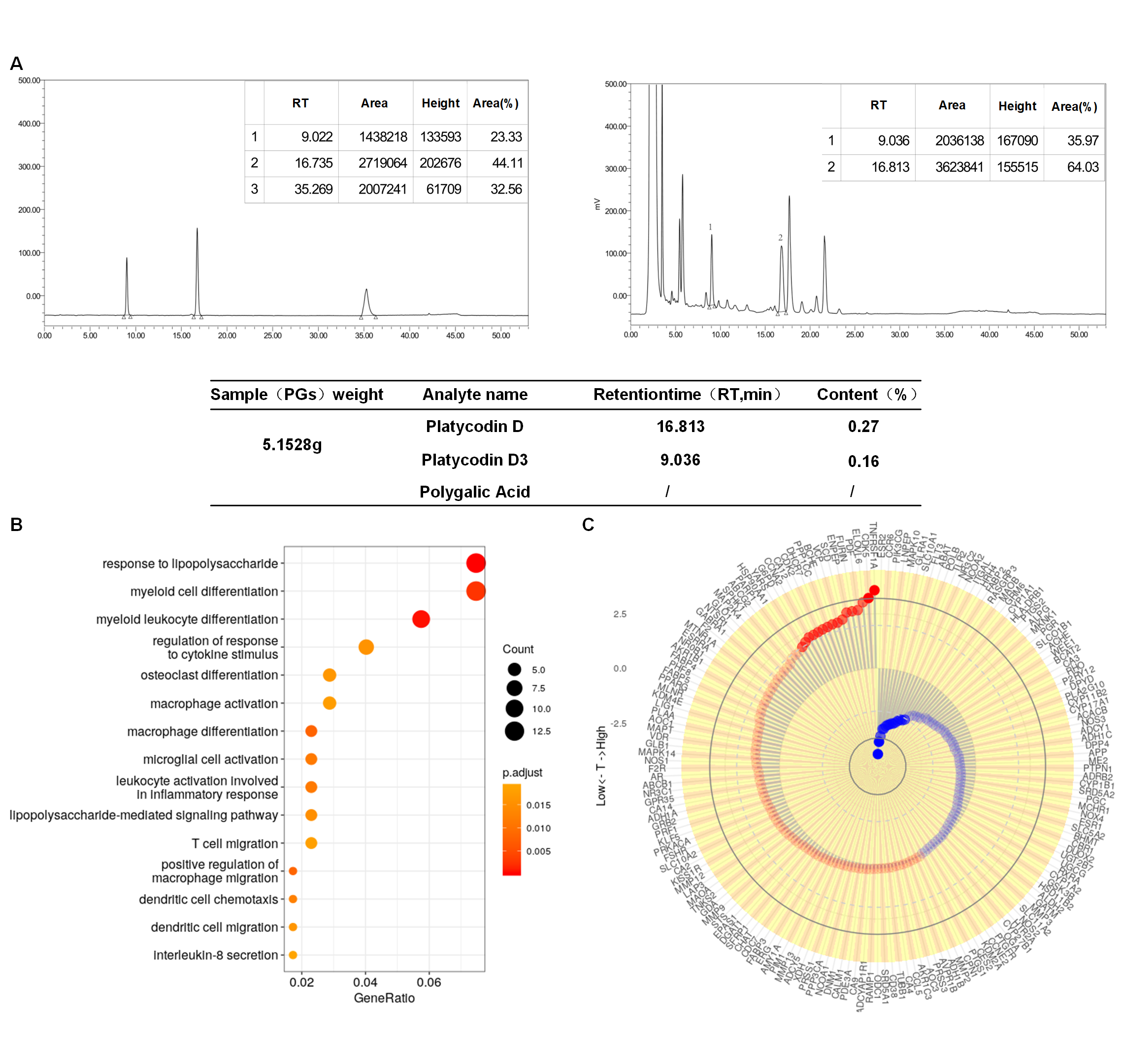

Supplement: Supplementary file 6 [file Image2.TIF]

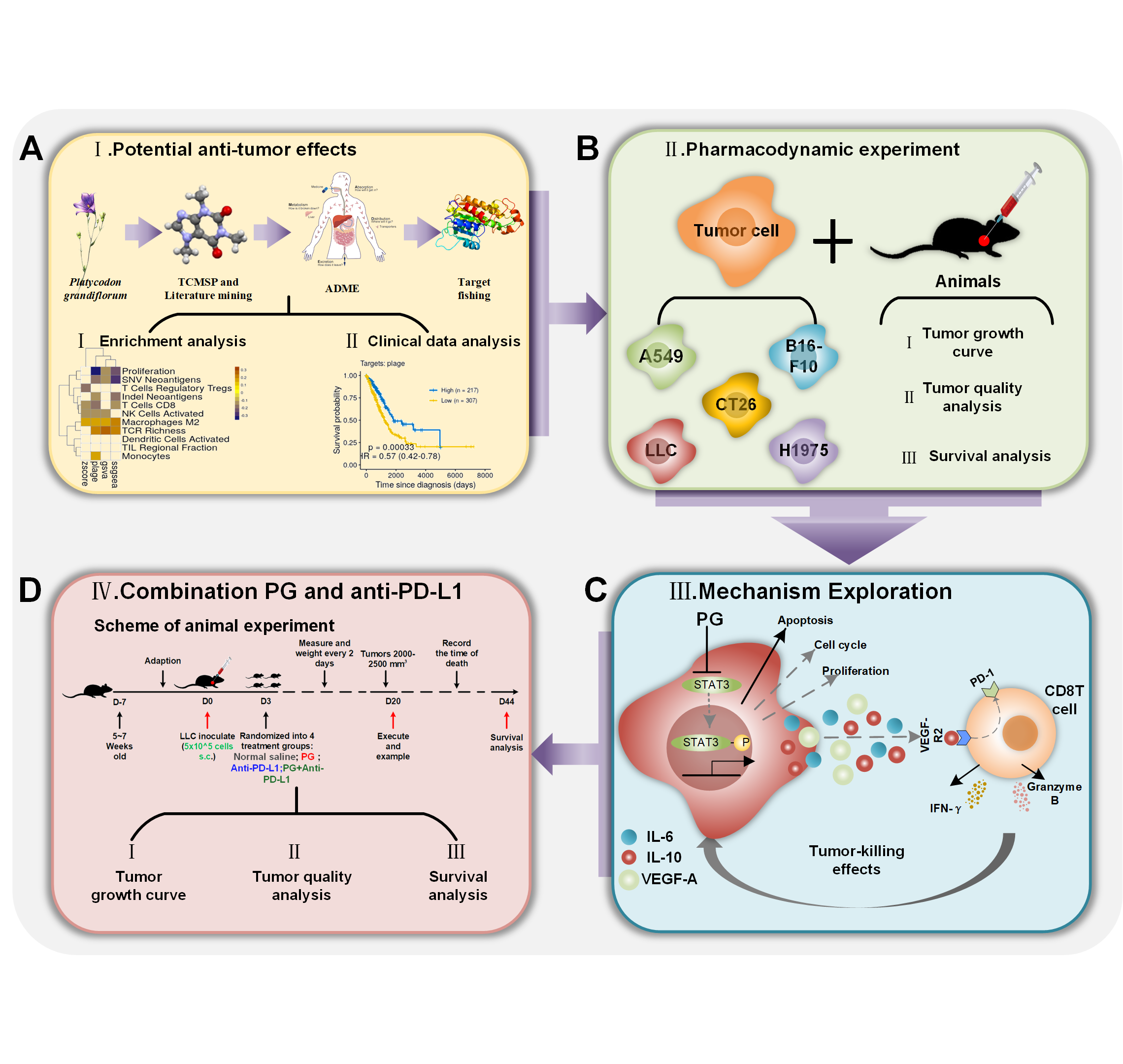

Supplement: Supplementary file 7 [file Image1.TIF]

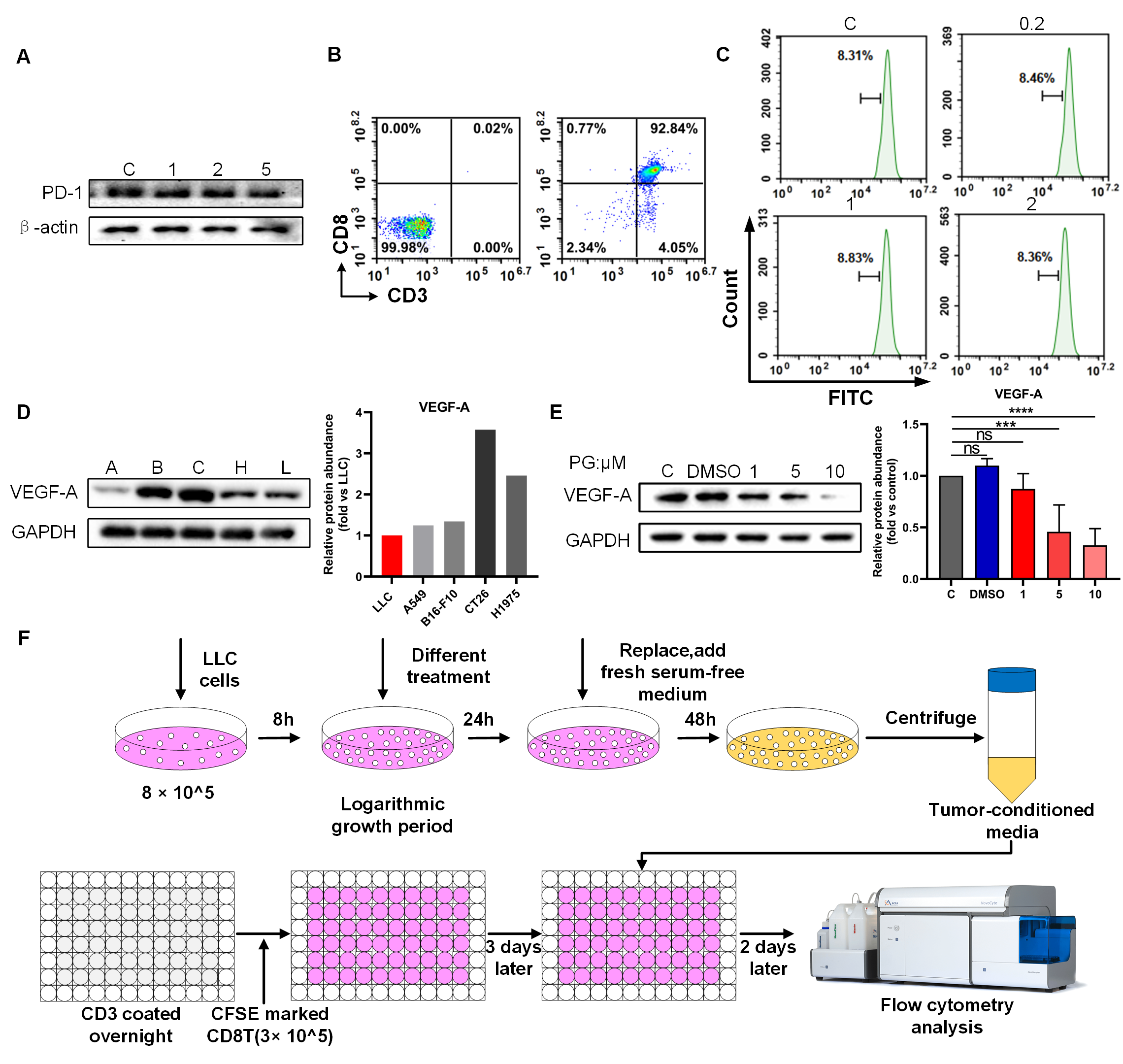

Supplement: Supplementary file 9 [file Image5.TIF]
