# Supplementary material for: Platycodon grandiflorum Triggers Antitumor Immunity by Restricting PD-1 Expression of CD8+ T Cells in Local Tumor Microenvironment
Source: Front Pharmacol. 2022 Apr 14;13:774440. doi: 10.3389/fphar.2022.774440 (PMC9046572; doi:10.3389/fphar.2022.774440)
Supplement: Supplementary file 3 [file Table1.DOCX]

| Mol | Molecule Name | OB | DL | HL | Structure | Number of targets |
| --- | --- | --- | --- | --- | --- | --- |
| M1 | Platycoside A | 5.81 | 0.01 | Long | 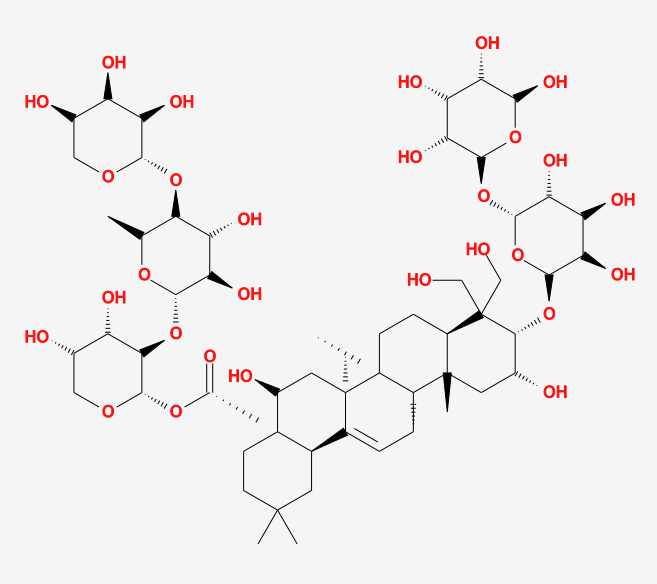 | 19 |
| M2 | Platycoside B | 2.20 | 0.02 | Long | 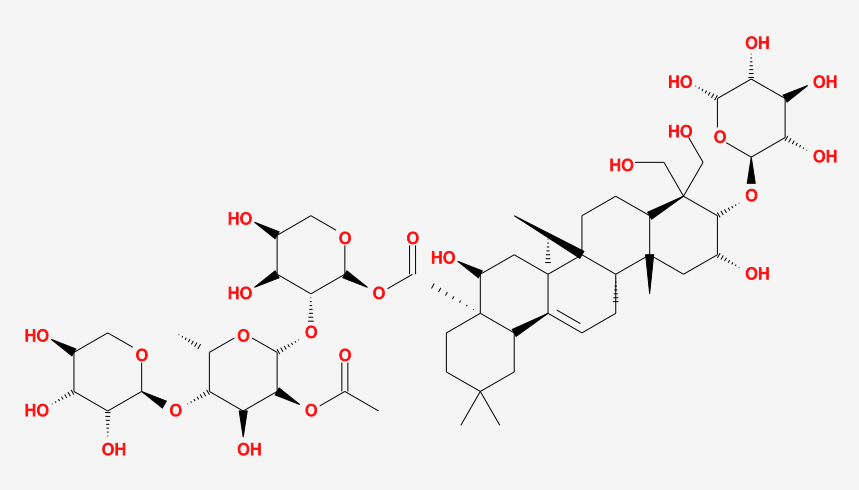 | 44 |
| M3 | Platycoside C | 3.39 | 0.02 | Long | 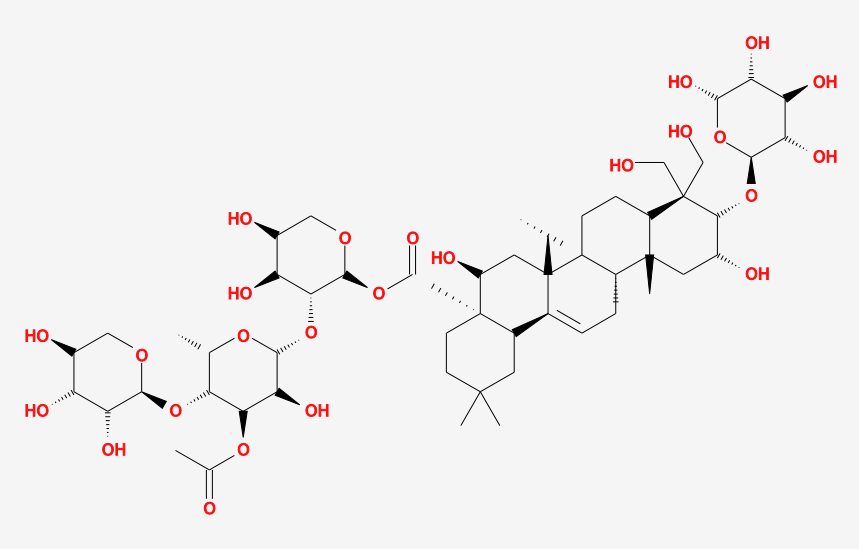 | 21 |
| M8 | Platycoside G2 | 6.84 | 0.01 | Long | 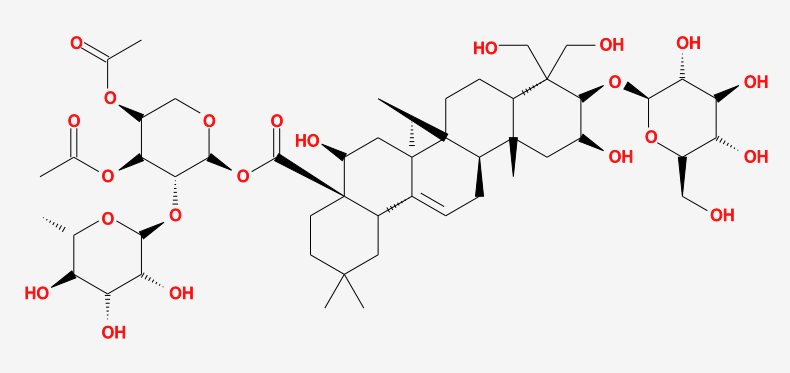 | 21 |
| M11 | Platycoside I | 7.44 | 0.00 | Long | 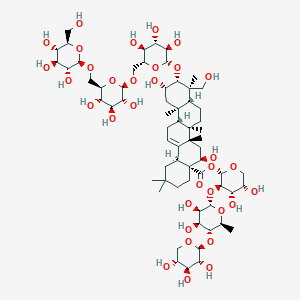 | 17 |
| M12 | Platycoside J | 3.10 | 0.03 | Long | 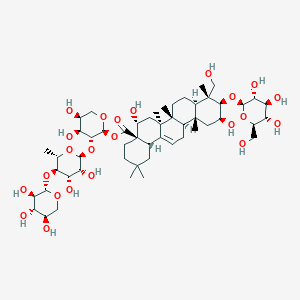 | 22 |
| M14 | Platycoside L | 2.11 | 0.11 | Long | 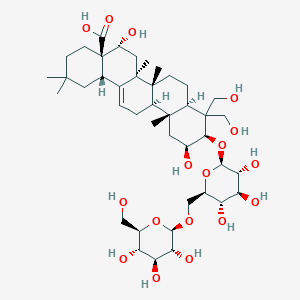 | 22 |
| M20 | Platycodin A | 7.71 | 0.01 | Long | 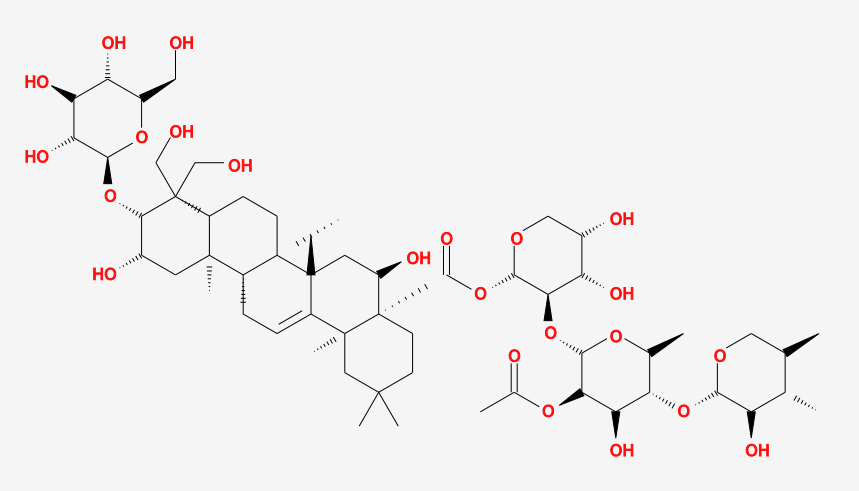 | 27 |
| M21 | Platycodin C | 7.61 | 0.01 | Long | 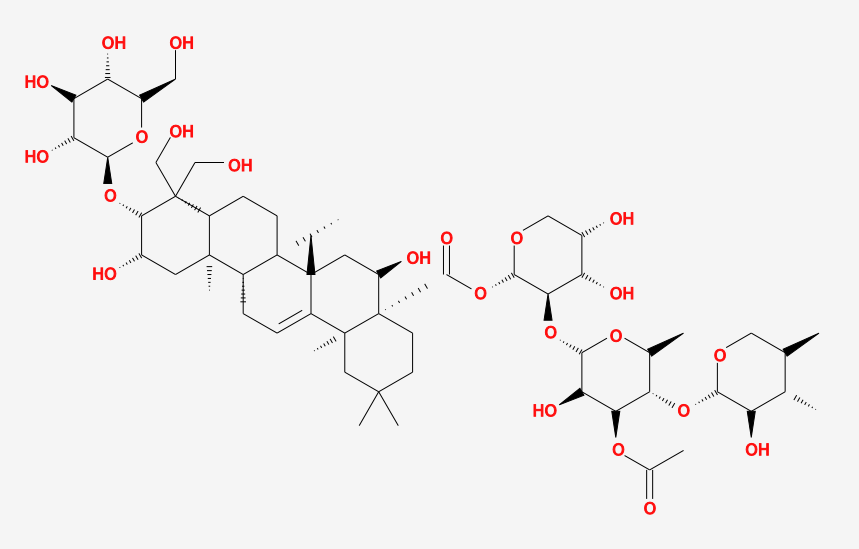 | 18 |
| M22 | Platycodin D | 7.58 | 0.01 | Long | 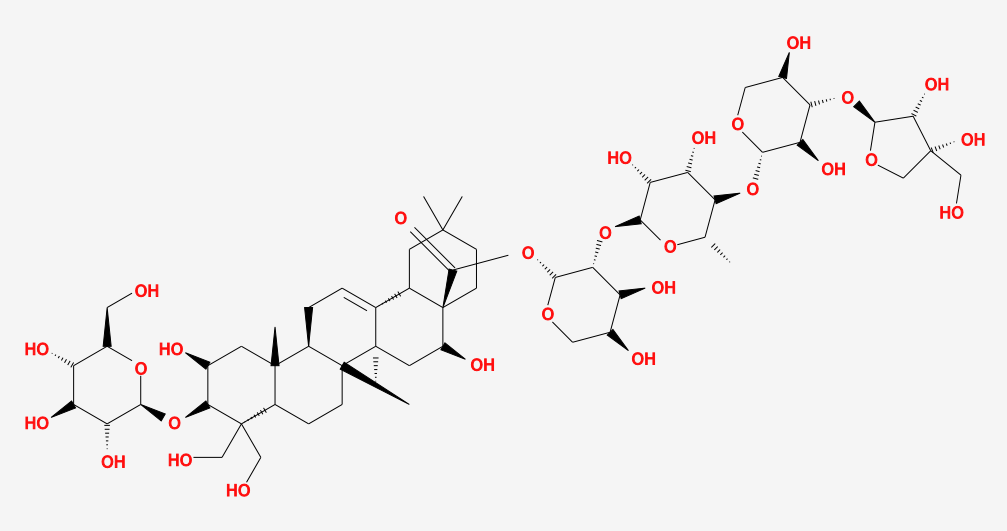 | 22 |
| M23 | Platycodin D2 | 7.57 | 0.00 | Long | 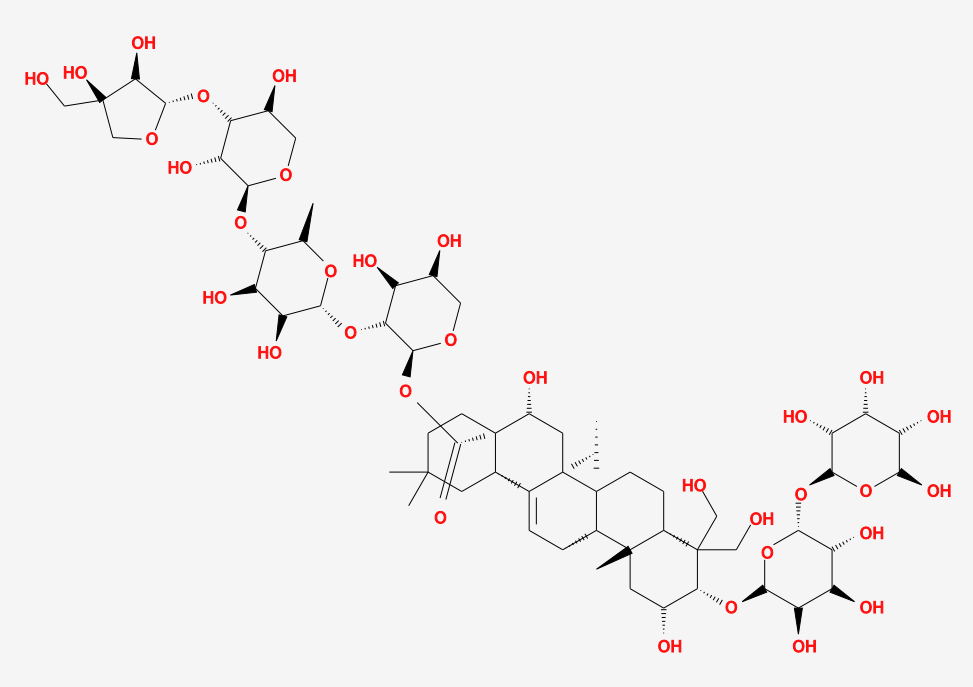 | 14 |
| M24 | Platycodin D3 | 7.57 | 0.00 | Long | 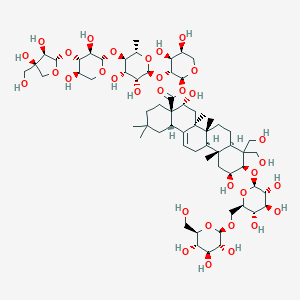 | 19 |
| M31 | 3'-O-acetylPlatycodin D2 | 7.57 | 0.00 | Short | 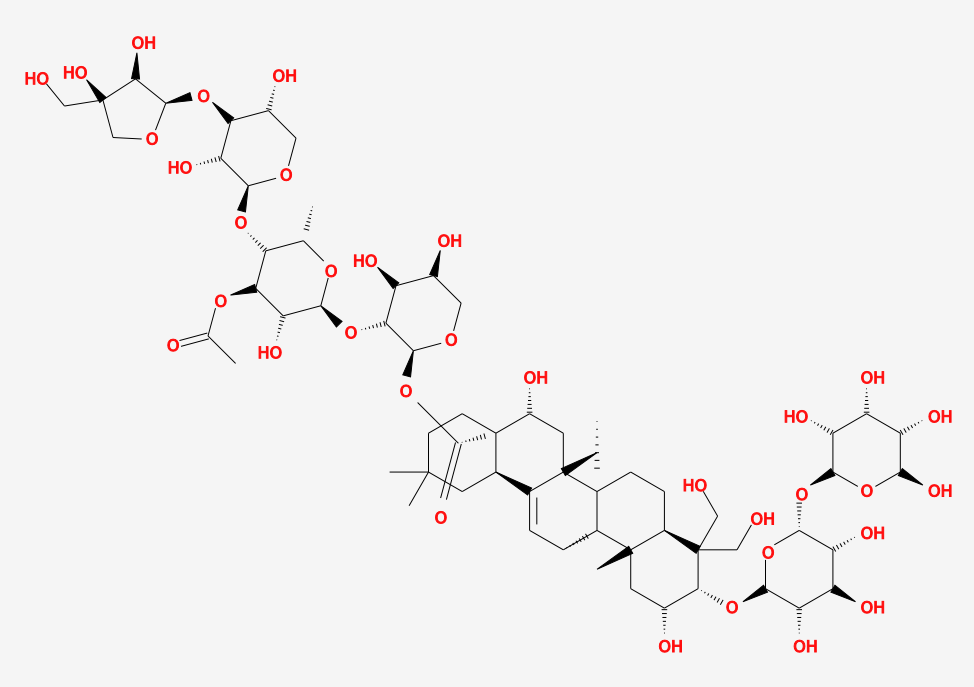 | 8 |
| M33 | 3-O-β-D-glucopyranosyl platycodigenin methyl ester | 13.77 | 0.29 | Long | 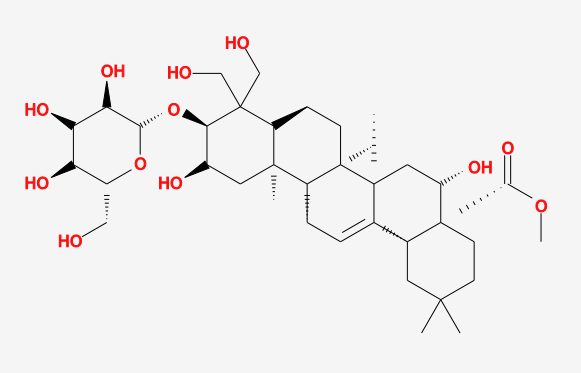 | 22 |
| M34 | 3-O-β-D-laminaribiosyl platycodigenin methyl ester | 2.80 | 0.11 | Long | 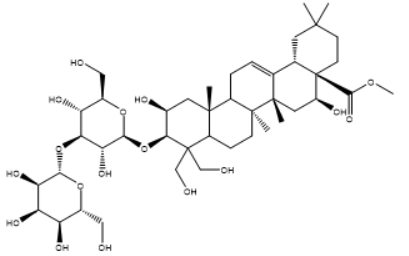 | 20 |
| M35 | 3-O-β-D-gentiobiosyl platycodigenin methyl ester | 4.88 | 0.11 | Long | 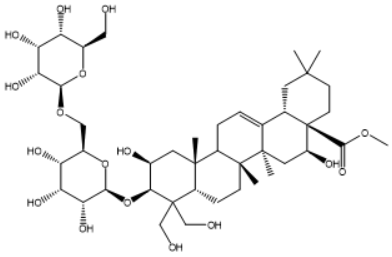 | 30 |
| M39 | platycodon B | 17.61 | 0.16 | Long | 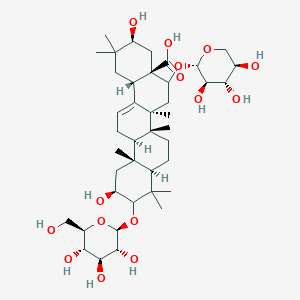 | 24 |
| M40 | Platycogenic acid A | 21.29 | 0.65 | Short | 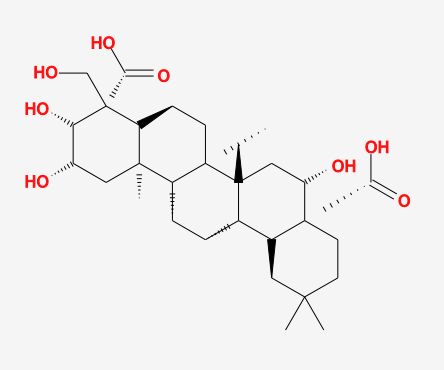 | 19 |
| M41 | Platycogenic acid B | 17.83 | 0.64 | Short | 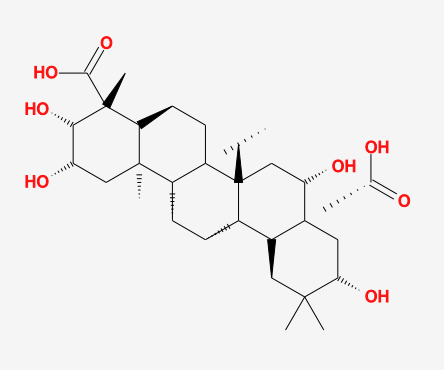 | 22 |
| M42 | Platycogenic acid C | 13.06 | 0.69 | Short | 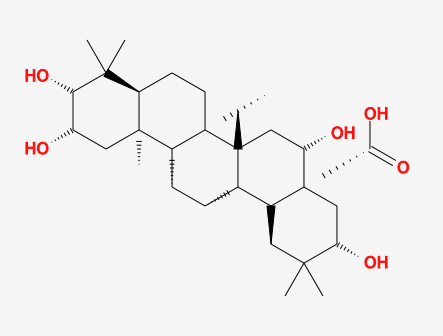 | 20 |
| M48 | Polygalacic acid | 11.06 | 0.70 | Short | 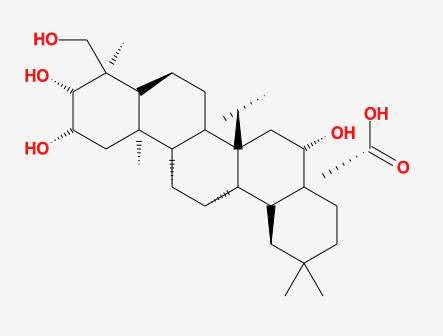 | 19 |
| M49 | platicodigenin | 6.36 | 0.67 | Short | 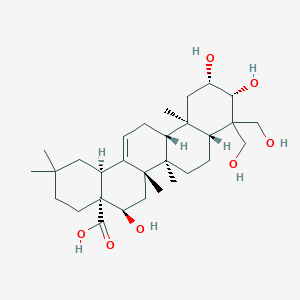 | 19 |
| M50 | 3''-O-Acetyl platyconic acid A | 7.73 | 0.01 | Long | 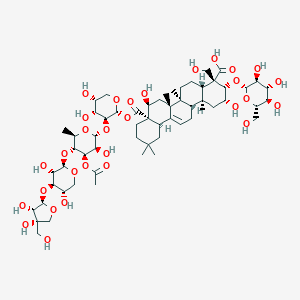 | 22 |
| M56 | beta-Sitosterol | 36.91 | 0.75 | Short | 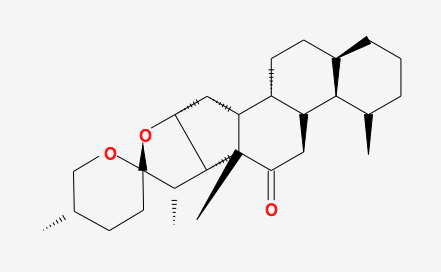 | 19 |
| M57 | Betulin | 16.22 | 0.78 | Long | 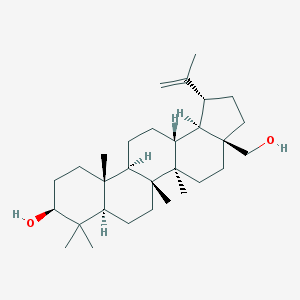 | 25 |
| M74 | 3-O-laminaribiosylplatycodigenin methyl ester | 5.97 | 0.65 | Short | 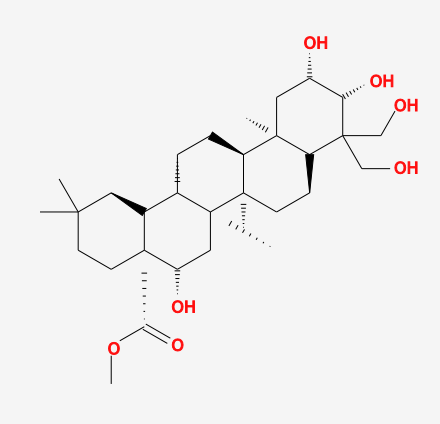 | 14 |
| M77 | 3-O-β-gentiobiosyl platycodigenin methylester | 13.50 | 0.11 | Long | 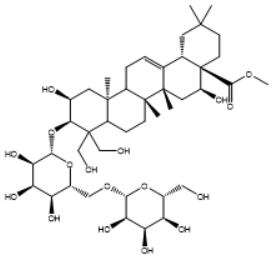 | 21 |
| M87 | dimethyl 2-O-methyl-3-O-a-D-glucopyranosyl platycogenate A | 39.21 | 0.25 | Long | 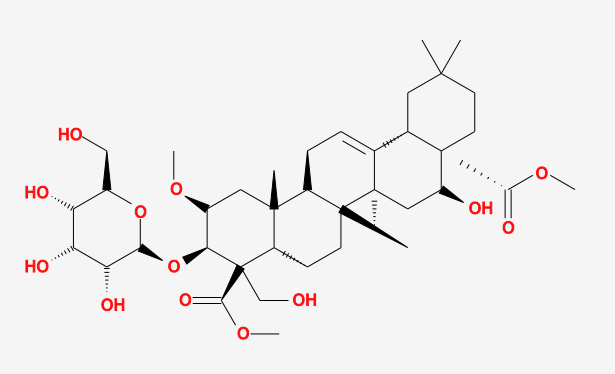 | 14 |
| M109 | nicotinic acid | 47.65 | 0.02 | Long | 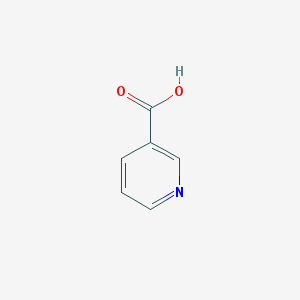 | 37 |
| M111 | Platycodigenin | 21.19 | 0.67 | Short | 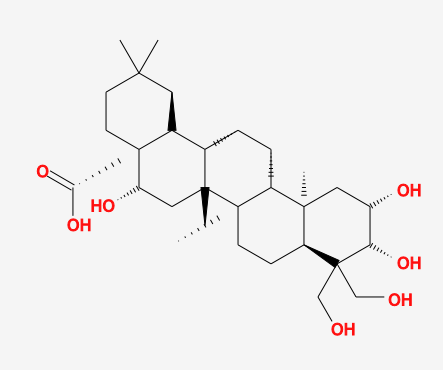 | 26 |
