# Supplementary material for: Platycodon grandiflorum Triggers Antitumor Immunity by Restricting PD-1 Expression of CD8+ T Cells in Local Tumor Microenvironment
Source: Front Pharmacol. 2022 Apr 14;13:774440. doi: 10.3389/fphar.2022.774440 (PMC9046572; doi:10.3389/fphar.2022.774440)
Supplement: Supplementary file 8 [file Table3.PDF]

UniProt-ID	Protein names	Gene names	Species	Degree
P11413	Glucose-6-phosphate 1-dehydrogenase	G6PD	Homo sapiens (Human)	22
Q9NRD8	Dual oxidase 2	DUOX2	Homo sapiens (Human)	20
P16662	UDP-glucuronosyltransferase 2B7	UGT2B7	Homo sapiens (Human)	18
P18405	3-oxo-5-alpha-steroid 4-dehydrogenase 1	SRD5A1	Homo sapiens (Human)	18
P36873	Serine/threonine-protein phosphatase PP1-gamma catalytic subunit	PPP1CC	Homo sapiens (Human)	18
P80365	Corticosteroid 11-beta-dehydrogenase isozyme 2	HSD11B2	Homo sapiens (Human)	18
Q08828	Adenylate cyclase type 1	ADCY1	Homo sapiens (Human)	18
Q8N474	Secreted frizzled-related protein 1	SFRP1	Homo sapiens (Human)	17
P04150	Glucocorticoid receptor	NR3C1	Homo sapiens (Human)	17
Q969F8	KiSS-1 receptor	KISS1R	Homo sapiens (Human)	17
Q9UBM7	7-dehydrocholesterol reductase	DHCR7	Homo sapiens (Human)	17
Q08209	Serine/threonine-protein phosphatase 2B catalytic subunit alpha isoform	PPP3CA	Homo sapiens (Human)	16
O60502	Protein O-GlcNAcase	OGA	Homo sapiens (Human)	16
P30989	Neurotensin receptor type 1	NTSR1	Homo sapiens (Human)	16
P09958	Furin	FURIN	Homo sapiens (Human)	14
P28329	Choline O-acetyltransferase	CHAT	Homo sapiens (Human)	14
O60894	Receptor activity-modifying protein 1	RAMP1	Homo sapiens (Human)	12
Q12908	Ileal sodium/bile acid cotransporter	SLC10A2	Homo sapiens (Human)	11
P13501	C-C motif chemokine 5	CCL5	Homo sapiens (Human)	11
Q99705	Melanin-concentrating hormone receptor 1	MCHR1	Homo sapiens (Human)	10

Q9NYA1	Sphingosine kinase 1	SPHK1	Homo sapiens (Human)	9
P23219	Prostaglandin G/H synthase 1	PTGS1	Homo sapiens (Human)	9
P35354	Prostaglandin G/H synthase 2	PTGS2	Homo sapiens (Human)	8
P35228	Nitric oxide synthase, inducible	NOS2	Homo sapiens (Human)	8
O75907	Diacylglycerol O-acyltransferase 1	DGAT1	Homo sapiens (Human)	7
P07477	Trypsin-1	PRSS1	Homo sapiens (Human)	6
P10636	Microtubule-associated protein tau	MAPT	Homo sapiens (Human)	6
P24941	Cyclin-dependent kinase 2	CDK2	Homo sapiens (Human)	6
Q86YN6	Peroxisome proliferator-activated receptor gamma coactivator 1-beta	PGC	Homo sapiens (Human)	5
P11473	Vitamin D3 receptor	VDR	Homo sapiens (Human)	5
Q13887	Krueppel-like factor 5	KLF5	Homo sapiens (Human)	5
P29474	Nitric oxide synthase	NOS3	Homo sapiens (Human)	5
P11309	Serine/threonine-protein kinase pim-1	PIM1	Homo sapiens (Human)	5
Q15596	Nuclear receptor coactivator 2	NCOA2	Homo sapiens (Human)	5
P00918	Carbonic anhydrase 2	CA2	Homo sapiens (Human)	5
P10275	Androgen receptor	AR	Homo sapiens (Human)	5
P30291	Wee1-like protein kinase	WEE1	Homo sapiens (Human)	4
Q9H5J4	Elongation of very long chain fatty acids protein 6	ELOVL6	Homo sapiens (Human)	4
Q9Y6L6	Solute carrier organic anion transporter family member 1B1	SLCO1B1	Homo sapiens (Human)	4
Q9UPP1	Histone lysine demethylase PHF8	PHF8	Homo sapiens (Human)	4
P07327	Alcohol dehydrogenase 1A	ADH1A	Homo sapiens (Human)	4

P00734	Prothrombin	F2	Homo sapiens (Human)	4
Q07075	Glutamyl aminopeptidase	ENPEP	Homo sapiens (Human)	4
P37231	Peroxisome proliferator-activated receptor gamma	PPARG	Homo sapiens (Human)	4
P07900	Heat shock protein HSP 90-alpha	HSP90AA1	Homo sapiens (Human)	4
P03372	Estrogen receptor	ESR1	Homo sapiens (Human)	4
P27487	Dipeptidyl peptidase 4	DPP4	Homo sapiens (Human)	4
P20248	Cyclin-A2	CCNA2	Homo sapiens (Human)	4
P05067	Amyloid-beta precursor protein	APP	Homo sapiens (Human)	4
P16278	Beta-galactosidase, EC	GLB1	Homo sapiens (Human)	3
Q05193	Dynamin-1	DNM1	Homo sapiens (Human)	3
Q93088	Betaine--homocysteine S-methyltransferase 1	BHMT	Homo sapiens (Human)	3
P22303	Acetylcholinesterase	ACHE	Homo sapiens (Human)	3
Q9Y2K7	Lysine-specific demethylase 2A	KDM2A	Homo sapiens (Human)	3
Q16853	Membrane primary amine oxidase	AOC3	Homo sapiens (Human)	3
P18031	Tyrosine-protein phosphatase non-receptor type 1	PTPN1	Homo sapiens (Human)	3
P29475	Nitric oxide synthase	NOS1	Homo sapiens (Human)	3
Q16539	Mitogen-activated protein kinase 14	MAPK14	Homo sapiens (Human)	3
Q92731	Estrogen receptor beta	ESR2	Homo sapiens (Human)	3
P04798	Cytochrome P450 1A1	CYP1A1	Homo sapiens (Human)	3
O00748	Cocaine esterase	CES2	Homo sapiens (Human)	3
O95622	Adenylate cyclase type 5	ADCY5	Homo sapiens (Human)	3

O43193	Motilin receptor	MLNR	Homo sapiens (Human)	2
P05093	Steroid 17-alpha-hydroxylase/17,20 lyase	CYP17A1	Homo sapiens (Human)	2
P31639	Sodium/glucose cotransporter 2	SLC5A2	Homo sapiens (Human)	2
Q9H244	P2Y purinoceptor 12	P2RY12	Homo sapiens (Human)	2
P25116	Proteinase-activated receptor 1	F2R	Homo sapiens (Human)	2
P53779	Mitogen-activated protein kinase 10	MAPK10	Homo sapiens (Human)	2
Q16678	Cytochrome P450 1B1	CYP1B1	Homo sapiens (Human)	2
P05177	Cytochrome P450 1A2	CYP1A2	Homo sapiens (Human)	2
P01857	Immunoglobulin heavy constant gamma 1	IGHG1	Homo sapiens (Human)	2
Q9BUB5	MAP kinase-interacting serine/threonine-protein kinase 1	MKNK1	Homo sapiens (Human)	2
Q9Y2T3	Guanine deaminase	GDA	Homo sapiens (Human)	2
P19801	Amiloride-sensitive amine oxidase	AOC1	Homo sapiens (Human)	2
P00326	Alcohol dehydrogenase 1C	ADH1C	Homo sapiens (Human)	2
P54577	Tyrosine--tRNA ligase	YARS	Homo sapiens (Human)	2
P28838	Cytosol aminopeptidase	LAP3	Homo sapiens (Human)	2
P18858	DNA ligase 1	LIG1	Homo sapiens (Human)	2
P14867	Gamma-aminobutyric acid receptor subunit alpha-1 receptor subunit alpha-1)	GABRA1	Homo sapiens (Human)	2
Q9H4B7	Tubulin beta-1 chain	TUBB1	Homo sapiens (Human)	2
P19438	Tumor necrosis factor receptor superfamily member 1A	TNFRSF1A	Homo sapiens (Human)	2

P51843	Nuclear receptor subfamily 0 group B member 1	NR0B1	Homo sapiens (Human)	2
P28223	5-hydroxytryptamine receptor 2A	HTR2A	Homo sapiens (Human)	2
P49841	Glycogen synthase kinase-3 beta	GSK3B	Homo sapiens (Human)	2
P51684	C-C chemokine receptor type 6	CCR6	Homo sapiens (Human)	2
P05091	Aldehyde dehydrogenase, mitochondrial	ALDH2	Homo sapiens (Human)	2
O15496	Group 10 secretory phospholipase A2	PLA2G10	Homo sapiens (Human)	1
P06746	DNA polymerase beta	POLB	Homo sapiens (Human)	1
Q9UIQ6	Leucyl-cystinyl aminopeptidase, Cystinyl aminopeptidase	LNPEP	Homo sapiens (Human)	1
P11308	Transcriptional regulator ERG	ERG	Homo sapiens (Human)	1
P20701	Integrin alpha-L	ITGAL	Homo sapiens (Human)	1
Q16790	Carbonic anhydrase 9	CA9	Homo sapiens (Human)	1
P22748	Carbonic anhydrase 4	CA4	Homo sapiens (Human)	1
P07451	Carbonic anhydrase 3	CA3	Homo sapiens (Human)	1
Q9ULX7	Carbonic anhydrase 14	CA14	Homo sapiens (Human)	1
O43570	Carbonic anhydrase 12	CA12	Homo sapiens (Human)	1
P23368	NAD-dependent malic enzyme	ME2	Homo sapiens (Human)	1
P27338	Amine oxidase [flavin-containing] B	MAOB	Homo sapiens (Human)	1
O15303	Metabotropic glutamate receptor 6	GRM6	Homo sapiens (Human)	1
P23415	Glycine receptor subunit alpha-1	GLRA1	Homo sapiens (Human)	1
P50440	Glycine amidinotransferase	GATM	Homo sapiens (Human)	1
P06276	Cholinesterase	BCHE	Homo sapiens (Human)	1

O15382	Branched-chain-amino-acid aminotransferase	BCAT2	Homo sapiens (Human)	1
P15121	Aldo-keto reductase family 1 member B1	AKR1B1	Homo sapiens (Human)	1
P80404	4-aminobutyrate aminotransferase	ABAT	Homo sapiens (Human)	1
P43088	Prostaglandin F2-alpha receptor	PTGFR	Homo sapiens (Human)	1
P01911	HLA class II histocompatibility antigen	HLA-DRB1	Homo sapiens (Human)	1
O00763	Acetyl-CoA carboxylase 2	ACACB	Homo sapiens (Human)	1
P55072	Transitional endoplasmic reticulum ATPase - ATPase p97 subunit)	VCP	Homo sapiens (Human)	1
Q9H3N8	Histamine H4 receptor, H4R	HRH4	Homo sapiens (Human)	1
P31213	3-oxo-5-alpha-steroid 4-dehydrogenase 2	SRD5A2	Homo sapiens (Human)	1
Q14973	Sodium/bile acid cotransporter /bile acid cotransporter) /taurocholate transport protein)	SLC10A1	Homo sapiens (Human)	1
P19793	Retinoic acid receptor RXR-alpha	RXRA	Homo sapiens (Human)	1
Q8IV61	Ras guanyl-releasing protein 3	RASGRP3	Homo sapiens (Human)	1
P06401	Progesterone receptor	PGR	Homo sapiens (Human)	1
P08235	Mineralocorticoid receptor	NR3C2	Homo sapiens (Human)	1
P23945	Follicle-stimulating hormone receptor	FSHR	Homo sapiens (Human)	1
O15528	25-hydroxyvitamin D-1 alpha hydroxylase	CYP27B1	Homo sapiens (Human)	1
Q16739	Ceramide glucosyltransferase	UGCG	Homo sapiens (Human)	1
O60603	Toll-like receptor 2	TLR2	Homo sapiens (Human)	1
O00767	Stearoyl-CoA desaturase	SCD	Homo sapiens (Human)	1
P11926	Ornithine decarboxylase	ODC1	Homo sapiens (Human)	1

P62993	Growth factor receptor-bound protein 2	GRB2	Homo sapiens (Human)	1
Q01469	Fatty acid-binding protein 5	FABP5	Homo sapiens (Human)	1
P15090	Fatty acid-binding protein, adipocyte	FABP4	Homo sapiens (Human)	1
P05413	Fatty acid-binding protein	FABP3	Homo sapiens (Human)	1
P12104	Fatty acid-binding protein, intestinal	FABP2	Homo sapiens (Human)	1
P47901	Vasopressin V1b receptor	AVPR1B	Homo sapiens (Human)	1
P41586	Pituitary adenylate cyclase-activating polypeptide type I receptor	ADCYAP1R1	Homo sapiens (Human)	1
P47989	Xanthine dehydrogenase	XDH	Homo sapiens (Human)	1
Q9H2K2	Poly [ADP-ribose] polymerase tankyrase-2	TNKS2	Homo sapiens (Human)	1
P09874	Poly [ADP-ribose] polymerase 1	PARP1	Homo sapiens (Human)	1
Q9NPH5	NADPH oxidase 4	NOX4	Homo sapiens (Human)	1
P14780	Matrix metalloproteinase-9	MMP9	Homo sapiens (Human)	1
P08254	Matrix metalloproteinase-3	MMP3	Homo sapiens (Human)	1
P08253	Matrix metalloproteinase-2	MMP2	Homo sapiens (Human)	1
P45452	Matrix metalloproteinase-13	MMP13	Homo sapiens (Human)	1
P39900	Matrix metalloproteinase-12	MMP12	Homo sapiens (Human)	1
P21397	Amine oxidase A	MAOA	Homo sapiens (Human)	1
Q15046	Lysine--tRNA ligase	KARS1	Homo sapiens (Human)	1
Q9HC97	G-protein coupled receptor 35	GPR35	Homo sapiens (Human)	1
Q04760	Lactoylglutathione lyase	GLO1	Homo sapiens (Human)	1
P36888	Receptor-type tyrosine-protein kinase FLT3	FLT3	Homo sapiens (Human)	1

Q00535	Cyclin-dependent-like kinase 5	CDK5	Homo sapiens (Human)	1
P28907	ADP-ribosyl cyclase/cyclic ADP-ribose hydrolase 1	CD38	Homo sapiens (Human)	1
O96020	G1/S-specific cyclin-E2	CCNE2	Homo sapiens (Human)	1
P0DUB6	Alpha-amylase 1A	AMY1A	Homo sapiens (Human)	1
Q9UNQ0	Broad substrate specificity ATP-binding cassette transporter ABCG2	ABCG2	Homo sapiens (Human)	1
P08183	ATP-dependent translocase ABCB1	ABCB1	Homo sapiens (Human)	1
P08100	Rhodopsin	RHO	Homo sapiens (Human)	1
P49281	Natural resistance-associated macrophage protein 2	SLC11A2	Homo sapiens (Human)	1
P35030	Trypsin-3	PRSS3	Homo sapiens (Human)	1
Q12882	Dihydropyrimidine dehydrogenase	DPYD	Homo sapiens (Human)	1
P19099	Cytochrome P450 11B2	CYP11B2	Homo sapiens (Human)	1
O00311	Cell division cycle 7-related protein kinase	CDC7	Homo sapiens (Human)	1
P00325	All-trans-retinol dehydrogenase ADH1B	ADH1B	Homo sapiens (Human)	1
Q8TF42	Ubiquitin-associated and SH3 domain-containing protein B	STS	Homo sapiens (Human)	1
Q9Y263	Phospholipase A-2-activating protein	PLAA	Homo sapiens (Human)	1
P48736	Phosphatidylinositol 4,5-bisphosphate 3-kinase catalytic subunit gamma isoform	PIK3CG	Homo sapiens (Human)	1
B2RXH2	Lysine-specific demethylase 4E	KDM4E	Homo sapiens (Human)	1
P16152	Carbonyl reductase 1	CBR1	Homo sapiens (Human)	1

P10696	Alkaline phosphatase, germ cell type	ALPG	Homo sapiens (Human)	1
Q9HBH1	Peptide deformylase, mitochondrial	PDF	Homo sapiens (Human)	1
Q14432	cGMP-inhibited 3',5'-cyclic phosphodiesterase A	PDE3A	Homo sapiens (Human)	1
P45985	Dual specificity mitogen-activated protein kinase kinase 4	MAP2K4	Homo sapiens (Human)	1
P15169	Carboxypeptidase N catalytic chain	CPN1	Homo sapiens (Human)	1
P42330	Aldo-keto reductase family 1 member C3	AKR1C3	Homo sapiens (Human)	1
P07550	Beta-2 adrenergic receptor	ADRB2	Homo sapiens (Human)	1
P17612	cAMP-dependent protein kinase catalytic subunit alpha	PRKACA	Homo sapiens (Human)	1
P14222	Perforin-1	PRF1	Homo sapiens (Human)	1
Q15788	Nuclear receptor coactivator 1	NCOA1	Homo sapiens (Human)	1
P48039	Melatonin receptor type 1A	MTNR1A	Homo sapiens (Human)	1
P11474	Steroid hormone receptor ERR1	ESRRA	Homo sapiens (Human)	1
Q99814	Endothelial PAS domain-containing protein 1	EPAS1	Homo sapiens (Human)	1
P0DP23	Calmodulin-1	CALM1	Homo sapiens (Human)	1
